# Supplementary material for: A systematic review and meta-analysis on achievement emotions, working memory and student-teacher relationship during second language learning in primary school
Source: PLoS One. 2026 May 26;21(5):e0350119. doi: 10.1371/journal.pone.0350119 (PMC13210231; doi:10.1371/journal.pone.0350119)
Supplement: S1 File — (DOCX) [file pone.0350119.s001.docx]

**Supporting information for**

***A systematic review and meta-analysis on achievement emotions, working memory and student-teacher relationship during L2 learning in primary school.***

1. **Additional information for Method section**
   1. **Define search strategy**

Our research question is:

how achievement emotions, working memory, and student-teacher relationship can influence (singularly or together) the performance of English as L2 in primary school students.

Therefore, our PEO presents several types of exposure, taken into consideration individually and jointly:

- P (Population): primary/elementary school students engaged in learning an L2
- E (Exposure): 3 types of exposure related to L2 learning, taken into consideration individually or jointly:

1. achievement emotions.
2. working memory.
3. Student-teacher relationship.

- O (Outcome): influence (positive, negative, neutral) of exposures on L2 performance on primary school students.

Regarding “L2 performance”, we decided not to specify the type of performance (writing, reading, etc.) to include all type of performance in L2 learning.

Before starting literature search, we planned the following actions:

1. We consulted Preprint databases (e.g.: PsyArXiv, Open Science Framework, PROSPERO) making sure that no one else is doing the same job as us.
2. We registered the idea on the Open Science Framework.
3. We agreed on the databases to be consulted:
   1. Web of Science (WOS)
   2. PsyArticle
   3. Scopus
   4. Pubmed
4. We defined inclusion and exclusion factors.
   1. **Search string improvement for teacher-student relationship**

**TEACHER-STUDENT RELATIONSHIP:**

TS=(“ relationship(s)” OR “closeness “ OR “attachment *” OR “warmth” OR “support *” OR “relatedness” OR “involvement” OR “affiliation” OR “affection” OR “empathy” OR “trust” OR “sensitivity” OR “responsive” OR “like/liking” OR “care/caring” OR “conflict” OR “neglect” OR “rejection” OR “dislike” OR “negativity” OR “anger” OR “concern”) AND TS=(“teacher” OR “student” OR “child” OR “pupil” OR “positive” OR “negative”)

The teacher-student relationship search string involved more attention, as it is not a construct identifiable with one (e.g.: learning) or two terms (e.g.: achievement emotions; working memory), but with a sentence composed of at least three terms (teacher, student, relationship) and different synonyms of each:

TS=(“ relationship(s)” OR “closeness “ OR “attachment *” OR “warmth” OR “support *” OR “relatedness” OR “involvement” OR “affiliation” OR “affection” OR “empathy” OR “trust” OR “sensitivity” OR “responsive” OR “like/liking” OR “care/caring” OR “conflict” OR “neglect” OR “rejection” OR “dislike” OR “negativity” OR “anger” OR “concern”) AND TS=(“teacher” OR “student” OR “child” OR “pupil” OR ~~“positive” OR “negative”~~)

We decided eliminating the concepts of positive or negative: maintaining the neutrality of the relationship (not making it explicit) allows us to welcome both.

| First term and synonyms | Second term and synonyms | Third term and synonyms (news in red) | String writing proposal |
| --- | --- | --- | --- |
| Teacher | Student  Child  Pupil | Relationship | Relat* |
|  |  | closeness | Close* |
|  |  | attachment | Attach* |
|  |  | warmth | Warm* |
|  |  | Support | Support* |
|  |  | relatedness (already included in Relat*) |  |
|  |  | involvement | Involv* |
|  |  | affiliation | Affiliate* |
|  |  | affection | Affect* |
|  |  | Empathy | Empat* |
|  |  | trust | Trust* |
|  |  | sensitivity | Sensit* |
|  |  | responsive | Respons* |
|  |  | like/liking | Lik* |
|  |  | care/caring | Car* |
|  |  | conflict | Conflict* |
|  |  | neglect | Neglect* |
|  |  | rejection | Reject* |
|  |  | dislike | Dislik* |
|  |  | negativity | Negat* |
|  |  | Anger | Ang* |
|  |  | concern | Concer* |
|  |  | Rapport* | |
|  |  | encourage* | |
|  |  | engag* | |
|  |  | motive* | |
|  |  | respect* | |
|  |  | “classroom manag*” | |
|  |  | communicat* | |
|  |  | conflict* | |
|  |  | hostil* | |
|  |  | Tens* | |
|  |  | disagr* | |
|  |  | critic* | |

Considering the concept of “teacher student relationship”, it can also be written as “**teacher-student** relationship” or even “teacher **and** student relationship” (not counting the use of the asterisk). They may seem trivial, but the quotation marks bring all the words together very meticulously. We solved as follow (see the part highlighted in green, then only the synonym of the relationship changes):

TS=(“teach* student* relat*” OR “teach*-student* relat*” OR “teach* and student* relat*” OR “teach*-child* relat*” OR “teach* child* relat*” OR “teach* and child* relat*” OR “teach*-pupil* relat*” OR “teach* pupil* relat*” OR “teach* and pupil* relat*” OR “teach* student* close*” OR “teach*-student* close*” OR “teach* and student* close*” OR “teach*-child* close*” OR “teach* child* close*” OR “teach* and child* close*” OR “teach*-pupil* close*” OR “teach* pupil* close*” OR “teach* and pupil* close*” OR “teach* student* attach*” OR “teach*-student* attach*” OR “teach* and student* attach*” OR “teach*-child* attach*” OR “teach* child* attach*” OR “teach* and child* attach*” OR “teach*-pupil* attach*” OR “teach* pupil* attach*” OR “teach* and pupil* attach*” OR “teach* student* warm*” OR “teach*-student* warm*” OR “teach* and student* warm*” OR “teach*-child* warm*” OR “teach* child* warm*” OR “teach* and child* warm*” OR “teach*-pupil* warm*” OR “teach* pupil* warm*” OR “teach* and pupil* warm*” OR “teach* student* support*” OR “teach*-student* support*” OR “teach* and student* support*” OR “teach*-child* support*” OR “teach* child* support*” OR “teach* and child* support*” OR “teach*-pupil* support*” OR “teach* pupil* support*” OR “teach* and pupil* support*” OR “teach* student* involv*” OR “teach*-student* involv*” OR “teach* and student* involv*” OR “teach*-child* involv*” OR “teach* child* involv*” OR “teach* and child* involv*” OR “teach*-pupil* involv*” OR “teach* pupil* involv*” OR “teach* and pupil* involv*” OR “teach* student* affiliat*” OR “teach*-student* affiliat*” OR “teach* and student* affiliat*” OR “teach*-child* affiliat*” OR “teach* child* affiliat*” OR “teach* and child* affiliat*” OR “teach*-pupil* affiliat*” OR “teach* pupil* affiliat*” OR “teach* and pupil* affiliat*” OR “teach* student* affect*” OR “teach*-student* affect*” OR “teach* and student* affect*” OR “teach*-child* affect*” OR “teach* child* affect*” OR “teach* and child* affect*” OR “teach*-pupil* affect*” OR “teach* pupil* affect*” OR “teach* and pupil* affect*” OR “teach* student* empat*” OR “teach*-student* empat*” OR “teach* and student* empat*” OR “teach*-child* empat*” OR “teach* child* empat*” OR “teach* and child* empat*” OR “teach*-pupil* empat*” OR “teach* pupil* empat*” OR “teach* and pupil* empat*” OR “teach* student* trust*” OR “teach*-student* trust*” OR “teach* and student* trust*” OR “teach*-child* trust*” OR “teach* child* trust*” OR “teach* and child* trust*” OR “teach*-pupil* trust*” OR “teach* pupil* trust*” OR “teach* and pupil* trust*” OR “teach* student* sensit*” OR “teach*-student* sensit*” OR “teach* and student* sensit*” OR “teach*-child* sensit*” OR “teach* child* sensit*” OR “teach* and child* sensit*” OR “teach*-pupil* sensit*” OR “teach* pupil* sensit*” OR “teach* and pupil* sensit*” OR “teach* student* respons*” OR “teach*-student* respons*” OR “teach* and student* respons*” OR “teach*-child* respons*” OR “teach* child* respons*” OR “teach* and child* respons*” OR “teach*-pupil* respons*” OR “teach* pupil* respons*” OR “teach* and pupil* respons*” OR “teach* student* lik*” OR “teach*-student* lik*” OR “teach* and student* lik*” OR “teach*-child* lik*” OR “teach* child* lik*” OR “teach* and child* lik*” OR “teach*-pupil* lik*” OR “teach* pupil* lik*” OR “teach* and pupil* lik*” OR “teach* student* dislik*” OR “teach*-student* dislik*” OR “teach* and student* dislik*” OR “teach*-child* dislik*” OR “teach* child* dislik*” OR “teach* and child* dislik*” OR “teach*-pupil* dislik*” OR “teach* pupil* dislik*” OR “teach* and pupil* dislik*” OR “teach* student* car*” OR “teach*-student* car*” OR “teach* and student* car*” OR “teach*-child* car*” OR “teach* child* car*” OR “teach* and child* car*” OR “teach*-pupil* car*” OR “teach* pupil* car*” OR “teach* and pupil* car*” OR “teach* student* conflict*” OR “teach*-student* conflict*” OR “teach* and student* conflict*” OR “teach*-child* conflict*” OR “teach* child* conflict*” OR “teach* and child* conflict*” OR “teach*-pupil* conflict*” OR “teach* pupil* conflict*” OR “teach* and pupil* conflict*” OR “teach* student* neglect*” OR “teach*-student* neglect*” OR “teach* and student* neglect*” OR “teach*-child* neglect*” OR “teach* child* neglect*” OR “teach* and child* neglect*” OR “teach*-pupil* neglect*” OR “teach* pupil* neglect*” OR “teach* and pupil* neglect*” OR “teach* student* reject*” OR “teach*-student* reject*” OR “teach* and student* reject*” OR “teach*-child* reject*” OR “teach* child* reject*” OR “teach* and child* reject*” OR “teach*-pupil* reject*” OR “teach* pupil* reject*” OR “teach* and pupil* reject*” OR “teach* student* negat*” OR “teach*-student* negat*” OR “teach* and student* negat*” OR “teach*-child* negat*” OR “teach* child* negat*” OR “teach* and child* negat*” OR “teach*-pupil* negat*” OR “teach* pupil* negat*” OR “teach* and pupil* negat*” OR “teach* student* ang*” OR “teach*-student* ang*” OR “teach* and student* ang*” OR “teach*-child* ang*” OR “teach* child* ang*” OR “teach* and child* ang*” OR “teach*-pupil* ang*” OR “teach* pupil* ang*” OR “teach* and pupil* ang*” OR “teach* student* concer*” OR “teach*-student* concer*” OR “teach* and student* concer*” OR “teach*-child* concer*” OR “teach* child* concer*” OR “teach* and child* concer*” OR “teach*-pupil* concer*” OR “teach* pupil* concer*” OR “teach* and pupil* concer*”)

However, it was not complete enough. It did not consider the possibility that the subjects of the relationship were inverted: student-teacher relationship, etc...

The parentheses ensured that all terms were considered, even in different positions:

TS=((teach*) and (student* or pupil* or child*) and (Relat* OR Close* OR Attach* OR Warm* OR Support* OR Involv* OR Affiliat* OR Affect* OR Empat* OR Trust* OR Sensit* OR Respons* OR Lik* OR Car* OR Conflict* OR Neglect* OR Reject* OR Dislik* OR Negat* OR Ang* OR Concer* OR Rapport* OR encourage* OR engag* OR motiv* OR respect* OR “classroom manag*”))

Link: <https://www.webofscience.com/wos/woscc/summary/651ba317-5d6e-41a8-bc16-1bbd3aac27b0-010de31c01/relevance/1>

The parentheses marked in red should include the terms into a single concept. However, the number of articles is over 300,000: we supposed some error in writing the string that increases the noise of irrelevant articles.

Therefore, we decided the string should be used in its entirety (the one with the first lines marked in green) adding the same terms in the same bracket, but with the inversion of the subjects (highlighted in yellow):

TS=(“teach* student* relat*” OR “teach*-student* relat*” OR “teach* and student* relat*” OR “teach*-child* relat*” OR “teach* child* relat*” OR “teach* and child* relat*” OR “teach*-pupil* relat*” OR “teach* pupil* relat*” OR “teach* and pupil* relat*” OR “teach* student* close*” OR “teach*-student* close*” OR “teach* and student* close*” OR “teach*-child* close*” OR “teach* child* close*” OR “teach* and child* close*” OR “teach*-pupil* close*” OR “teach* pupil* close*” OR “teach* and pupil* close*” OR “teach* student* attach*” OR “teach*-student* attach*” OR “teach* and student* attach*” OR “teach*-child* attach*” OR “teach* child* attach*” OR “teach* and child* attach*” OR “teach*-pupil* attach*” OR “teach* pupil* attach*” OR “teach* and pupil* attach*” OR “teach* student* warm*” OR “teach*-student* warm*” OR “teach* and student* warm*” OR “teach*-child* warm*” OR “teach* child* warm*” OR “teach* and child* warm*” OR “teach*-pupil* warm*” OR “teach* pupil* warm*” OR “teach* and pupil* warm*” OR “teach* student* support*” OR “teach*-student* support*” OR “teach* and student* support*” OR “teach*-child* support*” OR “teach* child* support*” OR “teach* and child* support*” OR “teach*-pupil* support*” OR “teach* pupil* support*” OR “teach* and pupil* support*” OR “teach* student* involv*” OR “teach*-student* involv*” OR “teach* and student* involv*” OR “teach*-child* involv*” OR “teach* child* involv*” OR “teach* and child* involv*” OR “teach*-pupil* involv*” OR “teach* pupil* involv*” OR “teach* and pupil* involv*” OR “teach* student* affiliat*” OR “teach*-student* affiliat*” OR “teach* and student* affiliat*” OR “teach*-child* affiliat*” OR “teach* child* affiliat*” OR “teach* and child* affiliat*” OR “teach*-pupil* affiliat*” OR “teach* pupil* affiliat*” OR “teach* and pupil* affiliat*” OR “teach* student* affect*” OR “teach*-student* affect*” OR “teach* and student* affect*” OR “teach*-child* affect*” OR “teach* child* affect*” OR “teach* and child* affect*” OR “teach*-pupil* affect*” OR “teach* pupil* affect*” OR “teach* and pupil* affect*” OR “teach* student* empat*” OR “teach*-student* empat*” OR “teach* and student* empat*” OR “teach*-child* empat*” OR “teach* child* empat*” OR “teach* and child* empat*” OR “teach*-pupil* empat*” OR “teach* pupil* empat*” OR “teach* and pupil* empat*” OR “teach* student* trust*” OR “teach*-student* trust*” OR “teach* and student* trust*” OR “teach*-child* trust*” OR “teach* child* trust*” OR “teach* and child* trust*” OR “teach*-pupil* trust*” OR “teach* pupil* trust*” OR “teach* and pupil* trust*” OR “teach* student* sensit*” OR “teach*-student* sensit*” OR “teach* and student* sensit*” OR “teach*-child* sensit*” OR “teach* child* sensit*” OR “teach* and child* sensit*” OR “teach*-pupil* sensit*” OR “teach* pupil* sensit*” OR “teach* and pupil* sensit*” OR “teach* student* respons*” OR “teach*-student* respons*” OR “teach* and student* respons*” OR “teach*-child* respons*” OR “teach* child* respons*” OR “teach* and child* respons*” OR “teach*-pupil* respons*” OR “teach* pupil* respons*” OR “teach* and pupil* respons*” OR “teach* student* lik*” OR “teach*-student* lik*” OR “teach* and student* lik*” OR “teach*-child* lik*” OR “teach* child* lik*” OR “teach* and child* lik*” OR “teach*-pupil* lik*” OR “teach* pupil* lik*” OR “teach* and pupil* lik*” OR “teach* student* dislik*” OR “teach*-student* dislik*” OR “teach* and student* dislik*” OR “teach*-child* dislik*” OR “teach* child* dislik*” OR “teach* and child* dislik*” OR “teach*-pupil* dislik*” OR “teach* pupil* dislik*” OR “teach* and pupil* dislik*” OR “teach* student* car*” OR “teach*-student* car*” OR “teach* and student* car*” OR “teach*-child* car*” OR “teach* child* car*” OR “teach* and child* car*” OR “teach*-pupil* car*” OR “teach* pupil* car*” OR “teach* and pupil* car*” OR “teach* student* conflict*” OR “teach*-student* conflict*” OR “teach* and student* conflict*” OR “teach*-child* conflict*” OR “teach* child* conflict*” OR “teach* and child* conflict*” OR “teach*-pupil* conflict*” OR “teach* pupil* conflict*” OR “teach* and pupil* conflict*” OR “teach* student* neglect*” OR “teach*-student* neglect*” OR “teach* and student* neglect*” OR “teach*-child* neglect*” OR “teach* child* neglect*” OR “teach* and child* neglect*” OR “teach*-pupil* neglect*” OR “teach* pupil* neglect*” OR “teach* and pupil* neglect*” OR “teach* student* reject*” OR “teach*-student* reject*” OR “teach* and student* reject*” OR “teach*-child* reject*” OR “teach* child* reject*” OR “teach* and child* reject*” OR “teach*-pupil* reject*” OR “teach* pupil* reject*” OR “teach* and pupil* reject*” OR “teach* student* negat*” OR “teach*-student* negat*” OR “teach* and student* negat*” OR “teach*-child* negat*” OR “teach* child* negat*” OR “teach* and child* negat*” OR “teach*-pupil* negat*” OR “teach* pupil* negat*” OR “teach* and pupil* negat*” OR “teach* student* ang*” OR “teach*-student* ang*” OR “teach* and student* ang*” OR “teach*-child* ang*” OR “teach* child* ang*” OR “teach* and child* ang*” OR “teach*-pupil* ang*” OR “teach* pupil* ang*” OR “teach* and pupil* ang*” OR “teach* student* concer*” OR “teach*-student* concer*” OR “teach* and student* concer*” OR “teach*-child* concer*” OR “teach* child* concer*” OR “teach* and child* concer*” OR “teach*-pupil* concer*” OR “teach* pupil* concer*” OR “teach* and pupil* concer*” OR “student* teach* relat*” OR “student*-teach* relat*” OR “student* and teach* relat*” OR “child* teach* relat*” OR “child* and teach* relat*” OR “pupil* teach* relat*” OR “pupil* and teach* relat*” OR “student* teach* close*” OR “student*-teach* close*” OR “student* and teach* close*” OR “child* teach* close*” OR “child* and teach* close*” OR “pupil* teach* close*” OR “pupil* and teach* close*” OR “student* teach* attach*” OR “student*-teach* attach*” OR “student* and teach* attach*” OR “child* teach* attach*” OR “child* and teach* attach*” OR “pupil* teach* attach*” OR “pupil* and teach* attach*” OR “student* teach* warm*” OR “student*-teach* warm*” OR “student* and teach* warm*” OR “child* teach* warm*” OR “child* and teach* warm*” OR “pupil* teach* warm*” OR “pupil* and teach* warm*” OR “student* teach* support*” OR “student*-teach* support*” OR “student* and teach* support*” OR “child* teach* support*” OR “child* and teach* support*” OR “pupil* teach* support*” OR “pupil* and teach* support*” OR “student* teach* involv*” OR “student*-teach* involv*” OR “student* and teach* involv*” OR “child* teach* involv*” OR “child* and teach* involv*” OR “pupil* teach* involv*” OR “pupil* and teach* involv*” OR “student* teach* affiliat*” OR “student*-teach* affiliat*” OR “student* and teach* affiliat*” OR “child* teach* affiliat*” OR “child* and teach* affiliat*” OR “pupil* teach* affiliat*” OR “pupil* and teach* affiliat*” OR “student* teach* affect*” OR “student*-teach* affect*” OR “student* and teach* affect*” OR “child* teach* affect*” OR “child* and teach* affect*” OR “pupil* teach* affect*” OR “pupil* and teach* affect*” OR “student* teach* empat*” OR “student*-teach* empat*” OR “student* and teach* empat*” OR “child* teach* empat*” OR “child* and teach* empat*” OR “pupil* teach* empat*” OR “pupil* and teach* empat*” OR “student* teach* trust*” OR “student*-teach* trust*” OR “student* and teach* trust*” OR “child* teach* trust*” OR “child* and teach* trust*” OR “pupil* teach* trust*” OR “pupil* and teach* trust*” OR “student* teach* sensit*” OR “student*-teach* sensit*” OR “student* and teach* sensit*” OR “child* teach* sensit*” OR “child* and teach* sensit*” OR “pupil* teach* sensit*” OR “pupil* and teach* sensit*” OR “student* teach* respons*” OR “student*-teach* respons*” OR “student* and teach* respons*” OR “child* teach* respons*” OR “child* and teach* respons*” OR “pupil* teach* respons*” OR “pupil* and teach* respons*” OR “student* teach* lik*” OR “student*-teach* lik*” OR “student* and teach* lik*” OR “child* teach* lik*” OR “child* and teach* lik*” OR “pupil* teach* lik*” OR “pupil* and teach* lik*” OR “student* teach* dislik*” OR “student*-teach* dislik*” OR “student* and teach* dislik*” OR “child* teach* dislik*” OR “child* and teach* dislik*” OR “pupil* teach* dislik*” OR “pupil* and teach* dislik*” OR “student* teach* car*” OR “student*-teach* car*” OR “student* and teach* car*” OR “child* teach* car*” OR “child* and teach* car*” OR “pupil* teach* car*” OR “pupil* and teach* car*” OR “student* teach* conflict*” OR “student*-teach* conflict*” OR “student* and teach* conflict*” OR “child* teach* conflict*” OR “child* and teach* conflict*” OR “pupil* teach* conflict*” OR “pupil* and teach* conflict*” OR “student* teach* neglect*” OR “student*-teach* neglect*” OR “student* and teach* neglect*” OR “child* teach* neglect*” OR “child* and teach* neglect*” OR “pupil* teach* neglect*” OR “pupil* and teach* neglect*” OR “student* teach* reject*” OR “student*-teach* reject*” OR “student* and teach* reject*” OR “child* teach* reject*” OR “child* and teach* reject*” OR “pupil* teach* reject*” OR “pupil* and teach* reject*” OR “student* teach* negat*” OR “student*-teach* negat*” OR “student* and teach* negat*” OR “child* teach* negat*” OR “child* and teach* negat*” OR “pupil* teach* negat*” OR “pupil* and teach* negat*” OR “student* teach* ang*” OR “student*-teach* ang*” OR “student* and teach* ang*” OR “child* teach* ang*” OR “child* and teach* ang*” OR “pupil* teach* ang*” OR “pupil* and teach* ang*” OR “student* teach* concer*” OR “student*-teach* concer*” OR “student* and teach* concer*” OR “child* teach* concer*” OR “child* and teach* concer*” OR “pupil* teach* concer*” OR “pupil* and teach* concer*” OR “teach* student* rapport*” OR “teach*-student* rapport*” OR “teach* and student* rapport*” OR “teach*-child* rapport*” OR “teach* child* rapport*” OR “teach* and child* rapport*” OR “teach*-pupil* rapport*” OR “teach* pupil* rapport*” OR “teach* and pupil* rapport*” OR “student* teach* rapport*” OR “student*-teach* rapport*” OR “student* and teach* rapport*” OR “child* teach* rapport*” OR “child* and teach* rapport*” OR “pupil* teach* rapport*” OR “pupil* and teach* rapport*” OR “teach* student* encourage*” OR “teach*-student* encourage*” OR “teach* and student* encourage*” OR “teach*-child* encourage*” OR “teach* child* encourage*” OR “teach* and child* encourage*” OR “teach*-pupil* encourage*” OR “teach* pupil* encourage*” OR “teach* and pupil* encourage*” OR “student* teach* encourage*” OR “student*-teach* encourage*” OR “student* and teach* encourage*” OR “child* teach* encourage*” OR “child* and teach* encourage*” OR “pupil* teach* encourage*” OR “pupil* and teach* encourage*” OR “teach* student* engag*” OR “teach*-student* engag*” OR “teach* and student* engag*” OR “teach*-child* engag*” OR “teach* child* engag*” OR “teach* and child* engag*” OR “teach*-pupil* engag*” OR “teach* pupil* engag*” OR “teach* and pupil* engag*”OR “student* teach* engag*” OR “student*-teach* engag*” OR “student* and teach* engag*” OR “child* teach* engag*” OR “child* and teach* engag*” OR “pupil* teach* engag*” OR “pupil* and teach* engag*” OR “teach* student* motiv*” OR “teach*-student* motiv*” OR “teach* and student* motiv*” OR “teach*-child* motiv*” OR “teach* child* motiv*” OR “teach* and child* motiv*” OR “teach*-pupil* motiv*” OR “teach* pupil* motiv*” OR “teach* and pupil* motiv*” OR “student* teach* motiv*” OR “student*-teach* motiv*” OR “student* and teach* motiv*” OR “child* teach* motiv*” OR “child* and teach* motiv*” OR “pupil* teach* motiv*” OR “pupil* and teach* motiv*” OR “teach* student* respect*” OR “teach*-student* respect*” OR “teach* and student* respect*” OR “teach*-child* respect*” OR “teach* child* respect*” OR “teach* and child* respect*” OR “teach*-pupil* respect*” OR “teach* pupil* respect*” OR “teach* and pupil* respect*” OR “student* teach* respect*” OR “student*-teach* respect*” OR “student* and teach* respect*” OR “child* teach* respect*” OR “child* and teach* respect*” OR “pupil* teach* respect*” OR “pupil* and teach* respect*” OR “teach* student* classroom manag*” OR “teach*-student* classroom manag*” OR “teach* and student* classroom manag*” OR “teach*-child* classroom manag*” OR “teach* child* classroom manag*” OR “teach* and child* classroom manag*” OR “teach*-pupil* classroom manag*” OR “teach* pupil* classroom manag*” OR “teach* and pupil* classroom manag*” OR “student* teach* classroom manag*” OR “student*-teach* classroom manag*” OR “student* and teach* classroom manag*” OR “child* teach* classroom manag*” OR “child* and teach* classroom manag*” OR “pupil* teach* classroom manag*” OR “pupil* and teach* classroom manag*” OR “teach* student* communicat*” OR “teach*-student* communicat*” OR “teach* and student* communicat*” OR “teach*-child* communicat*” OR “teach* child* communicat*” OR “teach* and child* communicat*” OR “teach*-pupil* communicat*” OR “teach* pupil* communicat*” OR “teach* and pupil* communicat*” OR “student* teach* communicat*” OR “student*-teach* communicat*” OR “student* and teach* communicat*” OR “child* teach* communicat*” OR “child* and teach* communicat*” OR “pupil* teach* communicat*” OR “pupil* and teach* communicat*” OR “teach* student* conflict*” OR “teach*-student* conflict*” OR “teach* and student* conflict*” OR “teach*-child* conflict*” OR “teach* child* conflict*” OR “teach* and child* conflict*” OR “teach*-pupil* conflict*” OR “teach* pupil* conflict*” OR “teach* and pupil* conflict*” OR “student* teach* conflict*” OR “student*-teach* conflict*” OR “student* and teach* conflict*” OR “child* teach* conflict*” OR “child* and teach* conflict*” OR “pupil* teach* conflict*” OR “pupil* and teach* conflict*” OR “teach* student* hostil*” OR “teach*-student* hostil*” OR “teach* and student* hostil*” OR “teach*-child* hostil*” OR “teach* child* hostil*” OR “teach* and child* hostil*” OR “teach*-pupil* hostil*” OR “teach* pupil* hostil*” OR “teach* and pupil* hostil*” OR “student* teach* hostil*” OR “student*-teach* hostil*” OR “student* and teach* hostil*” OR “child* teach* hostil*” OR “child* and teach* hostil*” OR “pupil* teach* hostil*” OR “pupil* and teach* hostil*” OR “teach* student* tens*” OR “teach*-student* tens*” OR “teach* and student* tens*” OR “teach*-child* tens*” OR “teach* child* tens*” OR “teach* and child* tens*” OR “teach*-pupil* tens*” OR “teach* pupil* tens*” OR “teach* and pupil* tens*” OR “student* teach* tens*” OR “student*-teach* tens*” OR “student* and teach* tens*” OR “child* teach* tens*” OR “child* and teach* tens*” OR “pupil* teach* tens*” OR “pupil* and teach* tens*” OR “teach* student* disagr*” OR “teach*-student* disagr*” OR “teach* and student* disagr*” OR “teach*-child* disagr*” OR “teach* child* disagr*” OR “teach* and child* disagr*” OR “teach*-pupil* disagr*” OR “teach* pupil* disagr*” OR “teach* and pupil* disagr*” OR “student* teach* disagr*” OR “student*-teach* disagr*” OR “student* and teach* disagr*” OR “child* teach* disagr*” OR “child* and teach* disagr*” OR “pupil* teach* disagr*” OR “pupil* and teach* disagr*” OR “teach* student* critic*” OR “teach*-student* critic*” OR “teach* and student* critic*” OR “teach*-child* critic*” OR “teach* child* critic*” OR “teach* and child* critic*” OR “teach*-pupil* critic*” OR “teach* pupil* critic*” OR “teach* and pupil* critic*” OR “student* teach* critic*” OR “student*-teach* critic*” OR “student* and teach* critic*” OR “child* teach* critic*” OR “child* and teach* critic*” OR “pupil* teach* critic*” OR “pupil* and teach* critic*”)

Link: <https://www.webofscience.com/wos/woscc/summary/3323aeeb-126d-4219-95e8-acda2c306d8d-010de30b0a/relevance/1>

- 1. **Search strings’ combinations**

We combined all strings, namely: how achievement emotions, working memory and the student-teacher relationship can jointly influence the performance of English as l2 in primary school students.

**Dependent variable (L2 learning):**

TS=(L2 OR “L2 learn*” OR “second language*” OR “second language learning” or “foreign language*” OR FL OR “Second Language Acquisition” OR SLA) **AND**

**Population:**

TS=(“primary school*” or “elementary school*” or “elementary student*” or “primary student*”) **AND**

**Achievement emotions:**

TS=(“anxiet*” OR “sham*” OR “ang*” OR “enjo*” OR “bor*” OR “hop*” OR “prid*” OR “joy*” OR “frustrat*” OR “relie*” OR “relax*” OR “content*” OR “disapp*” OR “sad*” OR “grat*” OR “achievement emot*” OR “academic emot*” OR “emot*”) **AND**

**Working memory:**

TS=(“working memor*” or WM or “immediate memor*” or “operant memor*” or “provisional memor*” or “short-term memor*” OR STM or “affective working memor*” or “emotional working memor*” or “working memor* for valenc* stimul*” or “working memor* for emot* stimul*”) **AND**

**Teacher-student relationship:**

TS=(“teach* student* relat*” OR “teach*-student* relat*” OR “teach* and student* relat*” OR “teach*-child* relat*” OR “teach* child* relat*” OR “teach* and child* relat*” OR “teach*-pupil* relat*” OR “teach* pupil* relat*” OR “teach* and pupil* relat*” OR “teach* student* close*” OR “teach*-student* close*” OR “teach* and student* close*” OR “teach*-child* close*” OR “teach* child* close*” OR “teach* and child* close*” OR “teach*-pupil* close*” OR “teach* pupil* close*” OR “teach* and pupil* close*” OR “teach* student* attach*” OR “teach*-student* attach*” OR “teach* and student* attach*” OR “teach*-child* attach*” OR “teach* child* attach*” OR “teach* and child* attach*” OR “teach*-pupil* attach*” OR “teach* pupil* attach*” OR “teach* and pupil* attach*” OR “teach* student* warm*” OR “teach*-student* warm*” OR “teach* and student* warm*” OR “teach*-child* warm*” OR “teach* child* warm*” OR “teach* and child* warm*” OR “teach*-pupil* warm*” OR “teach* pupil* warm*” OR “teach* and pupil* warm*” OR “teach* student* support*” OR “teach*-student* support*” OR “teach* and student* support*” OR “teach*-child* support*” OR “teach* child* support*” OR “teach* and child* support*” OR “teach*-pupil* support*” OR “teach* pupil* support*” OR “teach* and pupil* support*” OR “teach* student* involv*” OR “teach*-student* involv*” OR “teach* and student* involv*” OR “teach*-child* involv*” OR “teach* child* involv*” OR “teach* and child* involv*” OR “teach*-pupil* involv*” OR “teach* pupil* involv*” OR “teach* and pupil* involv*” OR “teach* student* affiliat*” OR “teach*-student* affiliat*” OR “teach* and student* affiliat*” OR “teach*-child* affiliat*” OR “teach* child* affiliat*” OR “teach* and child* affiliat*” OR “teach*-pupil* affiliat*” OR “teach* pupil* affiliat*” OR “teach* and pupil* affiliat*” OR “teach* student* affect*” OR “teach*-student* affect*” OR “teach* and student* affect*” OR “teach*-child* affect*” OR “teach* child* affect*” OR “teach* and child* affect*” OR “teach*-pupil* affect*” OR “teach* pupil* affect*” OR “teach* and pupil* affect*” OR “teach* student* empat*” OR “teach*-student* empat*” OR “teach* and student* empat*” OR “teach*-child* empat*” OR “teach* child* empat*” OR “teach* and child* empat*” OR “teach*-pupil* empat*” OR “teach* pupil* empat*” OR “teach* and pupil* empat*” OR “teach* student* trust*” OR “teach*-student* trust*” OR “teach* and student* trust*” OR “teach*-child* trust*” OR “teach* child* trust*” OR “teach* and child* trust*” OR “teach*-pupil* trust*” OR “teach* pupil* trust*” OR “teach* and pupil* trust*” OR “teach* student* sensit*” OR “teach*-student* sensit*” OR “teach* and student* sensit*” OR “teach*-child* sensit*” OR “teach* child* sensit*” OR “teach* and child* sensit*” OR “teach*-pupil* sensit*” OR “teach* pupil* sensit*” OR “teach* and pupil* sensit*” OR “teach* student* respons*” OR “teach*-student* respons*” OR “teach* and student* respons*” OR “teach*-child* respons*” OR “teach* child* respons*” OR “teach* and child* respons*” OR “teach*-pupil* respons*” OR “teach* pupil* respons*” OR “teach* and pupil* respons*” OR “teach* student* lik*” OR “teach*-student* lik*” OR “teach* and student* lik*” OR “teach*-child* lik*” OR “teach* child* lik*” OR “teach* and child* lik*” OR “teach*-pupil* lik*” OR “teach* pupil* lik*” OR “teach* and pupil* lik*” OR “teach* student* dislik*” OR “teach*-student* dislik*” OR “teach* and student* dislik*” OR “teach*-child* dislik*” OR “teach* child* dislik*” OR “teach* and child* dislik*” OR “teach*-pupil* dislik*” OR “teach* pupil* dislik*” OR “teach* and pupil* dislik*” OR “teach* student* car*” OR “teach*-student* car*” OR “teach* and student* car*” OR “teach*-child* car*” OR “teach* child* car*” OR “teach* and child* car*” OR “teach*-pupil* car*” OR “teach* pupil* car*” OR “teach* and pupil* car*” OR “teach* student* conflict*” OR “teach*-student* conflict*” OR “teach* and student* conflict*” OR “teach*-child* conflict*” OR “teach* child* conflict*” OR “teach* and child* conflict*” OR “teach*-pupil* conflict*” OR “teach* pupil* conflict*” OR “teach* and pupil* conflict*” OR “teach* student* neglect*” OR “teach*-student* neglect*” OR “teach* and student* neglect*” OR “teach*-child* neglect*” OR “teach* child* neglect*” OR “teach* and child* neglect*” OR “teach*-pupil* neglect*” OR “teach* pupil* neglect*” OR “teach* and pupil* neglect*” OR “teach* student* reject*” OR “teach*-student* reject*” OR “teach* and student* reject*” OR “teach*-child* reject*” OR “teach* child* reject*” OR “teach* and child* reject*” OR “teach*-pupil* reject*” OR “teach* pupil* reject*” OR “teach* and pupil* reject*” OR “teach* student* negat*” OR “teach*-student* negat*” OR “teach* and student* negat*” OR “teach*-child* negat*” OR “teach* child* negat*” OR “teach* and child* negat*” OR “teach*-pupil* negat*” OR “teach* pupil* negat*” OR “teach* and pupil* negat*” OR “teach* student* ang*” OR “teach*-student* ang*” OR “teach* and student* ang*” OR “teach*-child* ang*” OR “teach* child* ang*” OR “teach* and child* ang*” OR “teach*-pupil* ang*” OR “teach* pupil* ang*” OR “teach* and pupil* ang*” OR “teach* student* concer*” OR “teach*-student* concer*” OR “teach* and student* concer*” OR “teach*-child* concer*” OR “teach* child* concer*” OR “teach* and child* concer*” OR “teach*-pupil* concer*” OR “teach* pupil* concer*” OR “teach* and pupil* concer*” OR “student* teach* relat*” OR “student*-teach* relat*” OR “student* and teach* relat*” OR “child* teach* relat*” OR “child* and teach* relat*” OR “pupil* teach* relat*” OR “pupil* and teach* relat*” OR “student* teach* close*” OR “student*-teach* close*” OR “student* and teach* close*” OR “child* teach* close*” OR “child* and teach* close*” OR “pupil* teach* close*” OR “pupil* and teach* close*” OR “student* teach* attach*” OR “student*-teach* attach*” OR “student* and teach* attach*” OR “child* teach* attach*” OR “child* and teach* attach*” OR “pupil* teach* attach*” OR “pupil* and teach* attach*” OR “student* teach* warm*” OR “student*-teach* warm*” OR “student* and teach* warm*” OR “child* teach* warm*” OR “child* and teach* warm*” OR “pupil* teach* warm*” OR “pupil* and teach* warm*” OR “student* teach* support*” OR “student*-teach* support*” OR “student* and teach* support*” OR “child* teach* support*” OR “child* and teach* support*” OR “pupil* teach* support*” OR “pupil* and teach* support*” OR “student* teach* involv*” OR “student*-teach* involv*” OR “student* and teach* involv*” OR “child* teach* involv*” OR “child* and teach* involv*” OR “pupil* teach* involv*” OR “pupil* and teach* involv*” OR “student* teach* affiliat*” OR “student*-teach* affiliat*” OR “student* and teach* affiliat*” OR “child* teach* affiliat*” OR “child* and teach* affiliat*” OR “pupil* teach* affiliat*” OR “pupil* and teach* affiliat*” OR “student* teach* affect*” OR “student*-teach* affect*” OR “student* and teach* affect*” OR “child* teach* affect*” OR “child* and teach* affect*” OR “pupil* teach* affect*” OR “pupil* and teach* affect*” OR “student* teach* empat*” OR “student*-teach* empat*” OR “student* and teach* empat*” OR “child* teach* empat*” OR “child* and teach* empat*” OR “pupil* teach* empat*” OR “pupil* and teach* empat*” OR “student* teach* trust*” OR “student*-teach* trust*” OR “student* and teach* trust*” OR “child* teach* trust*” OR “child* and teach* trust*” OR “pupil* teach* trust*” OR “pupil* and teach* trust*” OR “student* teach* sensit*” OR “student*-teach* sensit*” OR “student* and teach* sensit*” OR “child* teach* sensit*” OR “child* and teach* sensit*” OR “pupil* teach* sensit*” OR “pupil* and teach* sensit*” OR “student* teach* respons*” OR “student*-teach* respons*” OR “student* and teach* respons*” OR “child* teach* respons*” OR “child* and teach* respons*” OR “pupil* teach* respons*” OR “pupil* and teach* respons*” OR “student* teach* lik*” OR “student*-teach* lik*” OR “student* and teach* lik*” OR “child* teach* lik*” OR “child* and teach* lik*” OR “pupil* teach* lik*” OR “pupil* and teach* lik*” OR “student* teach* dislik*” OR “student*-teach* dislik*” OR “student* and teach* dislik*” OR “child* teach* dislik*” OR “child* and teach* dislik*” OR “pupil* teach* dislik*” OR “pupil* and teach* dislik*” OR “student* teach* car*” OR “student*-teach* car*” OR “student* and teach* car*” OR “child* teach* car*” OR “child* and teach* car*” OR “pupil* teach* car*” OR “pupil* and teach* car*” OR “student* teach* conflict*” OR “student*-teach* conflict*” OR “student* and teach* conflict*” OR “child* teach* conflict*” OR “child* and teach* conflict*” OR “pupil* teach* conflict*” OR “pupil* and teach* conflict*” OR “student* teach* neglect*” OR “student*-teach* neglect*” OR “student* and teach* neglect*” OR “child* teach* neglect*” OR “child* and teach* neglect*” OR “pupil* teach* neglect*” OR “pupil* and teach* neglect*” OR “student* teach* reject*” OR “student*-teach* reject*” OR “student* and teach* reject*” OR “child* teach* reject*” OR “child* and teach* reject*” OR “pupil* teach* reject*” OR “pupil* and teach* reject*” OR “student* teach* negat*” OR “student*-teach* negat*” OR “student* and teach* negat*” OR “child* teach* negat*” OR “child* and teach* negat*” OR “pupil* teach* negat*” OR “pupil* and teach* negat*” OR “student* teach* ang*” OR “student*-teach* ang*” OR “student* and teach* ang*” OR “child* teach* ang*” OR “child* and teach* ang*” OR “pupil* teach* ang*” OR “pupil* and teach* ang*” OR “student* teach* concer*” OR “student*-teach* concer*” OR “student* and teach* concer*” OR “child* teach* concer*” OR “child* and teach* concer*” OR “pupil* teach* concer*” OR “pupil* and teach* concer*” OR “teach* student* rapport*” OR “teach*-student* rapport*” OR “teach* and student* rapport*” OR “teach*-child* rapport*” OR “teach* child* rapport*” OR “teach* and child* rapport*” OR “teach*-pupil* rapport*” OR “teach* pupil* rapport*” OR “teach* and pupil* rapport*” OR “student* teach* rapport*” OR “student*-teach* rapport*” OR “student* and teach* rapport*” OR “child* teach* rapport*” OR “child* and teach* rapport*” OR “pupil* teach* rapport*” OR “pupil* and teach* rapport*” OR “teach* student* encourage*” OR “teach*-student* encourage*” OR “teach* and student* encourage*” OR “teach*-child* encourage*” OR “teach* child* encourage*” OR “teach* and child* encourage*” OR “teach*-pupil* encourage*” OR “teach* pupil* encourage*” OR “teach* and pupil* encourage*” OR “student* teach* encourage*” OR “student*-teach* encourage*” OR “student* and teach* encourage*” OR “child* teach* encourage*” OR “child* and teach* encourage*” OR “pupil* teach* encourage*” OR “pupil* and teach* encourage*” OR “teach* student* engag*” OR “teach*-student* engag*” OR “teach* and student* engag*” OR “teach*-child* engag*” OR “teach* child* engag*” OR “teach* and child* engag*” OR “teach*-pupil* engag*” OR “teach* pupil* engag*” OR “teach* and pupil* engag*”OR “student* teach* engag*” OR “student*-teach* engag*” OR “student* and teach* engag*” OR “child* teach* engag*” OR “child* and teach* engag*” OR “pupil* teach* engag*” OR “pupil* and teach* engag*” OR “teach* student* motiv*” OR “teach*-student* motiv*” OR “teach* and student* motiv*” OR “teach*-child* motiv*” OR “teach* child* motiv*” OR “teach* and child* motiv*” OR “teach*-pupil* motiv*” OR “teach* pupil* motiv*” OR “teach* and pupil* motiv*” OR “student* teach* motiv*” OR “student*-teach* motiv*” OR “student* and teach* motiv*” OR “child* teach* motiv*” OR “child* and teach* motiv*” OR “pupil* teach* motiv*” OR “pupil* and teach* motiv*” OR “teach* student* respect*” OR “teach*-student* respect*” OR “teach* and student* respect*” OR “teach*-child* respect*” OR “teach* child* respect*” OR “teach* and child* respect*” OR “teach*-pupil* respect*” OR “teach* pupil* respect*” OR “teach* and pupil* respect*” OR “student* teach* respect*” OR “student*-teach* respect*” OR “student* and teach* respect*” OR “child* teach* respect*” OR “child* and teach* respect*” OR “pupil* teach* respect*” OR “pupil* and teach* respect*” OR “teach* student* classroom manag*” OR “teach*-student* classroom manag*” OR “teach* and student* classroom manag*” OR “teach*-child* classroom manag*” OR “teach* child* classroom manag*” OR “teach* and child* classroom manag*” OR “teach*-pupil* classroom manag*” OR “teach* pupil* classroom manag*” OR “teach* and pupil* classroom manag*” OR “student* teach* classroom manag*” OR “student*-teach* classroom manag*” OR “student* and teach* classroom manag*” OR “child* teach* classroom manag*” OR “child* and teach* classroom manag*” OR “pupil* teach* classroom manag*” OR “pupil* and teach* classroom manag*” OR “teach* student* communicat*” OR “teach*-student* communicat*” OR “teach* and student* communicat*” OR “teach*-child* communicat*” OR “teach* child* communicat*” OR “teach* and child* communicat*” OR “teach*-pupil* communicat*” OR “teach* pupil* communicat*” OR “teach* and pupil* communicat*” OR “student* teach* communicat*” OR “student*-teach* communicat*” OR “student* and teach* communicat*” OR “child* teach* communicat*” OR “child* and teach* communicat*” OR “pupil* teach* communicat*” OR “pupil* and teach* communicat*” OR “teach* student* conflict*” OR “teach*-student* conflict*” OR “teach* and student* conflict*” OR “teach*-child* conflict*” OR “teach* child* conflict*” OR “teach* and child* conflict*” OR “teach*-pupil* conflict*” OR “teach* pupil* conflict*” OR “teach* and pupil* conflict*” OR “student* teach* conflict*” OR “student*-teach* conflict*” OR “student* and teach* conflict*” OR “child* teach* conflict*” OR “child* and teach* conflict*” OR “pupil* teach* conflict*” OR “pupil* and teach* conflict*” OR “teach* student* hostil*” OR “teach*-student* hostil*” OR “teach* and student* hostil*” OR “teach*-child* hostil*” OR “teach* child* hostil*” OR “teach* and child* hostil*” OR “teach*-pupil* hostil*” OR “teach* pupil* hostil*” OR “teach* and pupil* hostil*” OR “student* teach* hostil*” OR “student*-teach* hostil*” OR “student* and teach* hostil*” OR “child* teach* hostil*” OR “child* and teach* hostil*” OR “pupil* teach* hostil*” OR “pupil* and teach* hostil*” OR “teach* student* tens*” OR “teach*-student* tens*” OR “teach* and student* tens*” OR “teach*-child* tens*” OR “teach* child* tens*” OR “teach* and child* tens*” OR “teach*-pupil* tens*” OR “teach* pupil* tens*” OR “teach* and pupil* tens*” OR “student* teach* tens*” OR “student*-teach* tens*” OR “student* and teach* tens*” OR “child* teach* tens*” OR “child* and teach* tens*” OR “pupil* teach* tens*” OR “pupil* and teach* tens*” OR “teach* student* disagr*” OR “teach*-student* disagr*” OR “teach* and student* disagr*” OR “teach*-child* disagr*” OR “teach* child* disagr*” OR “teach* and child* disagr*” OR “teach*-pupil* disagr*” OR “teach* pupil* disagr*” OR “teach* and pupil* disagr*” OR “student* teach* disagr*” OR “student*-teach* disagr*” OR “student* and teach* disagr*” OR “child* teach* disagr*” OR “child* and teach* disagr*” OR “pupil* teach* disagr*” OR “pupil* and teach* disagr*” OR “teach* student* critic*” OR “teach*-student* critic*” OR “teach* and student* critic*” OR “teach*-child* critic*” OR “teach* child* critic*” OR “teach* and child* critic*” OR “teach*-pupil* critic*” OR “teach* pupil* critic*” OR “teach* and pupil* critic*” OR “student* teach* critic*” OR “student*-teach* critic*” OR “student* and teach* critic*” OR “child* teach* critic*” OR “child* and teach* critic*” OR “pupil* teach* critic*” OR “pupil* and teach* critic*”)

- 1. **Search process**

The following links and number of results referred on searches carried out on 22/11/2024

- - 1. Searches in preprint databases

PsyArxiv and Open Science Framework: No pre-recorded secondary work like ours was highlighted.

- - 1. Searches in the main databases

**Web of Science:**

- L2 + Population + Achievement Emotions: **481** results from Web of Science ([Https://Www.Webofscience.Com/Wos/Woscc/Summary/Cceab0ac-78dc-4640-893a-9ede9ea3ce5b-010ed9ea71/Relevance/1](https://www.webofscience.com/wos/woscc/summary/cceab0ac-78dc-4640-893a-9ede9ea3ce5b-010ed9ea71/relevance/1))
- L2 + Population + Working Memory: **46** results from Web of Science ([Https://Www.Webofscience.Com/Wos/Woscc/Summary/C905afc7-31e3-4640-9b16-B802d24d6e7f-010ed9ffc3/Relevance/1](https://www.webofscience.com/wos/woscc/summary/c905afc7-31e3-4640-9b16-b802d24d6e7f-010ed9ffc3/relevance/1))
- L2 + Population + Student-Teacher Relationship: **5** results from Web of Science ([Https://Www.Webofscience.Com/Wos/Woscc/Summary/Bbca79a8-4892-4e6c-8dc9-Cd7a66244654-010eda10ba/Relevance/1](https://www.webofscience.com/wos/woscc/summary/bbca79a8-4892-4e6c-8dc9-cd7a66244654-010eda10ba/relevance/1))
- L2 + Population + Achievement Emotions + Working Memory: **7** results from Web of Science ([Https://Www.Webofscience.Com/Wos/Woscc/Summary/5007224b-3dc2-4000-Bda7-B4f44257e97b-010eda569c/Relevance/1](https://www.webofscience.com/wos/woscc/summary/5007224b-3dc2-4000-bda7-b4f44257e97b-010eda569c/relevance/1))
- L2 + Population + Achievement Emotions + Student-Teacher Relationship: **1** results from Web of Science ([Https://Www.Webofscience.Com/Wos/Woscc/Summary/71fc724a-3e29-42c0-8be4-28a1981b3d5f-010eda697c/Relevance/1](https://www.webofscience.com/wos/woscc/summary/71fc724a-3e29-42c0-8be4-28a1981b3d5f-010eda697c/relevance/1))
- L2 + Population + Working Memory + Student-Teacher Relationship: **0** results
- L2 + Population + Achievement Emotions + Working Memory + Student-Teacher Relationship: **0** results

We inserted the terms of inclusion and exclusion:

- L2 + Population + Achievement Emotions: **44** results from Web of Science ([Https://Www.Webofscience.Com/Wos/Woscc/Summary/1b7f7bbe-Eddb-4964-A88d-8f5f036a5f65-0125d4eb07/Relevance/1](https://www.webofscience.com/wos/woscc/summary/1b7f7bbe-eddb-4964-a88d-8f5f036a5f65-0125d4eb07/relevance/1))
- L2 + Population + Working Memory: **41** results from Web of Science ([Https://Www.Webofscience.Com/Wos/Woscc/Summary/2172d76f-Dce3-49f9-8d7d-6f255579c490-0125d545f2/Relevance/1](https://www.webofscience.com/wos/woscc/summary/2172d76f-dce3-49f9-8d7d-6f255579c490-0125d545f2/relevance/1))
- L2 + Population + Achievement Emotions + Working Memory: **7** results from Web of Science ([Https://Www.Webofscience.Com/Wos/Woscc/Summary/5007224b-3dc2-4000-Bda7-B4f44257e97b-010eda569c/Relevance/1](https://www.webofscience.com/wos/woscc/summary/5007224b-3dc2-4000-bda7-b4f44257e97b-010eda569c/relevance/1))
- L2 + Population + Achievement Emotions + Student-Teacher Relationship: **1** results from Web of Science ([Https://Www.Webofscience.Com/Wos/Woscc/Summary/71fc724a-3e29-42c0-8be4-28a1981b3d5f-010eda697c/Relevance/1](https://www.webofscience.com/wos/woscc/summary/71fc724a-3e29-42c0-8be4-28a1981b3d5f-010eda697c/relevance/1))
- L2 + Population + Working Memory + Student-Teacher Relationship: **0** results
- L2 + Population + Achievement Emotions + Working Memory + Student-Teacher Relationship: **0** results

**Scopus:**

- L2 + Population + Achievement Emotions: **57** results from Scopus

https://www.scopus.com/results/results.uri?sort=plf-f&src=s&st1=%28L2+OR+%22L2+learn*%22+OR+%22second+language*%22+OR+%22second+language+learning%22+or+%22foreign+language*%22+OR+FL+OR+%22Second+Language+Acquisition%22+OR+SLA%29+AND+%28%22primary+school*%22+or+%22elementary+school*%22+or+%22elementary+student*%22+or+%22primary+student*%22%29+AND+%28%22anxiet*%22+OR+%22sham*%22+OR+%22ang*%22+OR+%22enjo*%22+OR+%22bor*%22+OR+%22hop*%22+OR+%22prid*%22+OR+%22joy*%22+OR+%22frustrat*%22+OR+%22relie*%22+OR+%22relax*%22+OR+%22content*%22+OR+%22disapp*%22+OR+%22sad*%22+OR+%22grat*%22+OR+%22achievement+emot*%22+OR+%22academic+emot*%22+OR+%22emot*%22%29&sid=4a07926c0e04f13fb264f4a3e46ef1d4&sot=b&sdt=cl&sl=480&s=TITLE-ABS-KEY%28%28L2+OR+%22L2+learn*%22+OR+%22second+language*%22+OR+%22second+language+learning%22+or+%22foreign+language*%22+OR+FL+OR+%22Second+Language+Acquisition%22+OR+SLA%29+AND+%28%22primary+school*%22+or+%22elementary+school*%22+or+%22elementary+student*%22+or+%22primary+student*%22%29+AND+%28%22anxiet*%22+OR+%22sham*%22+OR+%22ang*%22+OR+%22enjo*%22+OR+%22bor*%22+OR+%22hop*%22+OR+%22prid*%22+OR+%22joy*%22+OR+%22frustrat*%22+OR+%22relie*%22+OR+%22relax*%22+OR+%22content*%22+OR+%22disapp*%22+OR+%22sad*%22+OR+%22grat*%22+OR+%22achievement+emot*%22+OR+%22academic+emot*%22+OR+%22emot*%22%29%29&origin=resultslist&editSaveSearch=&yearFrom=Before+1960&yearTo=Present&sessionSearchId=4a07926c0e04f13fb264f4a3e46ef1d4&limit=10&cluster=scosubjabbr%2C%22PSYC%22%2Ct%2Bscolang%2C%22English%22%2Ct

- L2 + Population + Working Memory: **32** results from Scopus

https://www.scopus.com/results/results.uri?sort=plf-f&src=s&st1=%28L2+OR+%22L2+learn*%22+OR+%22second+language*%22+OR+%22second+language+learning%22+or+%22foreign+language*%22+OR+FL+OR+%22Second+Language+Acquisition%22+OR+SLA%29+AND+%28%22primary+school*%22+or+%22elementary+school*%22+or+%22elementary+student*%22+or+%22primary+student*%22%29+AND+%28%22working+memor*%22+or+WM+or+%22immediate+memor*%22+or+%22operant+memor*%22+or+%22provisional+memor*%22+or+%22short-term+memor*%22+OR+STM+or+%22affective+working+memor*%22+or+%22emotional+working+memor*%22+or+%22working+memor*+for+valenc*+stimul*%22+or+%22working+memor*+for+emot*+stimul*%22%29&sid=69eb829a6cc7954837e17436e74cf8e1&sot=b&sdt=cl&sl=513&s=TITLE-ABS-KEY%28%28L2+OR+%22L2+learn*%22+OR+%22second+language*%22+OR+%22second+language+learning%22+or+%22foreign+language*%22+OR+FL+OR+%22Second+Language+Acquisition%22+OR+SLA%29+AND+%28%22primary+school*%22+or+%22elementary+school*%22+or+%22elementary+student*%22+or+%22primary+student*%22%29+AND+%28%22working+memor*%22+or+WM+or+%22immediate+memor*%22+or+%22operant+memor*%22+or+%22provisional+memor*%22+or+%22short-term+memor*%22+OR+STM+or+%22affective+working+memor*%22+or+%22emotional+working+memor*%22+or+%22working+memor*+for+valenc*+stimul*%22+or+%22working+memor*+for+emot*+stimul*%22%29%29&origin=resultslist&editSaveSearch=&yearFrom=Before+1960&yearTo=Present&sessionSearchId=69eb829a6cc7954837e17436e74cf8e1&limit=10&cluster=scolang%2C%22English%22%2Ct

- L2 + Population + Student-Teacher Relationship: **5** results from Scopus

https://www.scopus.com/results/results.uri?sort=plf-f&src=s&st1=%28L2+OR+%22L2+learn*%22+OR+%22second+language*%22+OR+%22second+language+learning%22+or+%22foreign+language*%22+OR+FL+OR+%22Second+Language+Acquisition%22+OR+SLA%29+AND+%28%22primary+school*%22+or+%22elementary+school*%22+or+%22elementary+student*%22+or+%22primary+student*%22%29+AND+%28%22teach*+student*+relat*%22+OR+%22teach*-student*+relat*%22+OR+%22teach*+and+student*+relat*%22+OR+%22teach*-child*+relat*%22+OR+%22teach*+child*+relat*%22+OR+%22teach*+and+child*+relat*%22+OR+%22teach*-pupil*+relat*%22+OR+%22teach*+pupil*+relat*%22+OR+%22teach*+and+pupil*+relat*%22+OR+%22teach*+student*+close*%22+OR+%22teach*-student*+close*%22+OR+%22teach*+and+student*+close*%22+OR+%22teach*-child*+close*%22+OR+%22teach*+child*+close*%22+OR+%22teach*+and+child*+close*%22+OR+%22teach*-pupil*+close*%22+OR+%22teach*+pupil*+close*%22+OR+%22teach*+and+pupil*+close*%22+OR+%22teach*+student*+attach*%22+OR+%22teach*-student*+attach*%22+OR+%22teach*+and+student*+attach*%22+OR+%22teach*-child*+attach*%22+OR+%22teach*+child*+attach*%22+OR+%22teach*+and+child*+attach*%22+OR+%22teach*-pupil*+attach*%22+OR+%22teach*+pupil*+attach*%22+OR+%22teach*+and+pupil*+attach*%22+OR+%22teach*+student*+warm*%22+OR+%22teach*-student*+warm*%22+OR+%22teach*+and+student*+warm*%22+OR+%22teach*-child*+warm*%22+OR+%22teach*+child*+warm*%22+OR+%22teach*+and+child*+warm*%22+OR+%22teach*-pupil*+warm*%22+OR+%22teach*+pupil*+warm*%22+OR+%22teach*+and+pupil*+warm*%22+OR+%22teach*+student*+support*%22+OR+%22teach*-student*+support*%22+OR+%22teach*+and+student*+support*%22+OR+%22teach*-child*+support*%22+OR+%22teach*+child*+support*%22+OR+%22teach*+and+child*+support*%22+OR+%22teach*-pupil*+support*%22+OR+%22teach*+pupil*+support*%22+OR+%22teach*+and+pupil*+support*%22+OR+%22teach*+student*+involv*%22+OR+%22teach*-student*+involv*%22+OR+%22teach*+and+student*+involv*%22+OR+%22teach*-child*+involv*%22+OR+%22teach*+child*+involv*%22+OR+%22teach*+and+child*+involv*%22+OR+%22teach*-pupil*+involv*%22+OR+%22teach*+pupil*+involv*%22+OR+%22teach*+and+pupil*+involv*%22+OR+%22teach*+student*+affiliat*%22+OR+%22teach*-student*+affiliat*%22+OR+%22teach*+and+student*+affiliat*%22+OR+%22teach*-child*+affiliat*%22+OR+%22teach*+child*+affiliat*%22+OR+%22teach*+and+child*+affiliat*%22+OR+%22teach*-pupil*+affiliat*%22+OR+%22teach*+pupil*+affiliat*%22+OR+%22teach*+and+pupil*+affiliat*%22+OR+%22teach*+student*+affect*%22+OR+%22teach*-student*+affect*%22+OR+%22teach*+and+student*+affect*%22+OR+%22teach*-child*+affect*%22+OR+%22teach*+child*+affect*%22+OR+%22teach*+and+child*+affect*%22+OR+%22teach*-pupil*+affect*%22+OR+%22teach*+pupil*+affect*%22+OR+%22teach*+and+pupil*+affect*%22+OR+%22teach*+student*+empat*%22+OR+%22teach*-student*+empat*%22+OR+%22teach*+and+student*+empat*%22+OR+%22teach*-child*+empat*%22+OR+%22teach*+child*+empat*%22+OR+%22teach*+and+child*+empat*%22+OR+%22teach*-pupil*+empat*%22+OR+%22teach*+pupil*+empat*%22+OR+%22teach*+and+pupil*+empat*%22+OR+%22teach*+student*+trust*%22+OR+%22teach*-student*+trust*%22+OR+%22teach*+and+student*+trust*%22+OR+%22teach*-child*+trust*%22+OR+%22teach*+child*+trust*%22+OR+%22teach*+and+child*+trust*%22+OR+%22teach*-pupil*+trust*%22+OR+%22teach*+pupil*+trust*%22+OR+%22teach*+and+pupil*+trust*%22+OR+%22teach*+student*+sensit*%22+OR+%22teach*-student*+sensit*%22+OR+%22teach*+and+student*+sensit*%22+OR+%22teach*-child*+sensit*%22+OR+%22teach*+child*+sensit*%22+OR+%22teach*+and+child*+sensit*%22+OR+%22teach*-pupil*+sensit*%22+OR+%22teach*+pupil*+sensit*%22+OR+%22teach*+and+pupil*+sensit*%22+OR+%22teach*+student*+respons*%22+OR+%22teach*-student*+respons*%22+OR+%22teach*+and+student*+respons*%22+OR+%22teach*-child*+respons*%22+OR+%22teach*+child*+respons*%22+OR+%22teach*+and+child*+respons*%22+OR+%22teach*-pupil*+respons*%22+OR+%22teach*+pupil*+respons*%22+OR+%22teach*+and+pupil*+respons*%22+OR+%22teach*+student*+lik*%22+OR+%22teach*-student*+lik*%22+OR+%22teach*+and+student*+lik*%22+OR+%22teach*-child*+lik*%22+OR+%22teach*+child*+lik*%22+OR+%22teach*+and+child*+lik*%22+OR+%22teach*-pupil*+lik*%22+OR+%22teach*+pupil*+lik*%22+OR+%22teach*+and+pupil*+lik*%22+OR+%22teach*+student*+dislik*%22+OR+%22teach*-student*+dislik*%22+OR+%22teach*+and+student*+dislik*%22+OR+%22teach*-child*+dislik*%22+OR+%22teach*+child*+dislik*%22+OR+%22teach*+and+child*+dislik*%22+OR+%22teach*-pupil*+dislik*%22+OR+%22teach*+pupil*+dislik*%22+OR+%22teach*+and+pupil*+dislik*%22+OR+%22teach*+student*+car*%22+OR+%22teach*-student*+car*%22+OR+%22teach*+and+student*+car*%22+OR+%22teach*-child*+car*%22+OR+%22teach*+child*+car*%22+OR+%22teach*+and+child*+car*%22+OR+%22teach*-pupil*+car*%22+OR+%22teach*+pupil*+car*%22+OR+%22teach*+and+pupil*+car*%22+OR+%22teach*+student*+conflict*%22+OR+%22teach*-student*+conflict*%22+OR+%22teach*+and+student*+conflict*%22+OR+%22teach*-child*+conflict*%22+OR+%22teach*+child*+conflict*%22+OR+%22teach*+and+child*+conflict*%22+OR+%22teach*-pupil*+conflict*%22+OR+%22teach*+pupil*+conflict*%22+OR+%22teach*+and+pupil*+conflict*%22+OR+%22teach*+student*+neglect*%22+OR+%22teach*-student*+neglect*%22+OR+%22teach*+and+student*+neglect*%22+OR+%22teach*-child*+neglect*%22+OR+%22teach*+child*+neglect*%22+OR+%22teach*+and+child*+neglect*%22+OR+%22teach*-pupil*+neglect*%22+OR+%22teach*+pupil*+neglect*%22+OR+%22teach*+and+pupil*+neglect*%22+OR+%22teach*+student*+reject*%22+OR+%22teach*-student*+reject*%22+OR+%22teach*+and+student*+reject*%22+OR+%22teach*-child*+reject*%22+OR+%22teach*+child*+reject*%22+OR+%22teach*+and+child*+reject*%22+OR+%22teach*-pupil*+reject*%22+OR+%22teach*+pupil*+reject*%22+OR+%22teach*+and+pupil*+reject*%22+OR+%22teach*+student*+negat*%22+OR+%22teach*-student*+negat*%22+OR+%22teach*+and+student*+negat*%22+OR+%22teach*-child*+negat*%22+OR+%22teach*+child*+negat*%22+OR+%22teach*+and+child*+negat*%22+OR+%22teach*-pupil*+negat*%22+OR+%22teach*+pupil*+negat*%22+OR+%22teach*+and+pupil*+negat*%22+OR+%22teach*+student*+ang*%22+OR+%22teach*-student*+ang*%22+OR+%22teach*+and+student*+ang*%22+OR+%22teach*-child*+ang*%22+OR+%22teach*+child*+ang*%22+OR+%22teach*+and+child*+ang*%22+OR+%22teach*-pupil*+ang*%22+OR+%22teach*+pupil*+ang*%22+OR+%22teach*+and+pupil*+ang*%22+OR+%22teach*+student*+concer*%22+OR+%22teach*-student*+concer*%22+OR+%22teach*+and+student*+concer*%22+OR+%22teach*-child*+concer*%22+OR+%22teach*+child*+concer*%22+OR+%22teach*+and+child*+concer*%22+OR+%22teach*-pupil*+concer*%22+OR+%22teach*+pupil*+concer*%22+OR+%22teach*+and+pupil*+concer*%22+OR+%22student*+teach*+relat*%22+OR+%22student*-teach*+relat*%22+OR+%22student*+and+teach*+relat*%22+OR+%22child*+teach*+relat*%22+OR+%22child*+and+teach*+relat*%22+OR+%22pupil*+teach*+relat*%22+OR+%22pupil*+and+teach*+relat*%22+OR+%22student*+teach*+close*%22+OR+%22student*-teach*+close*%22+OR+%22student*+and+teach*+close*%22+OR+%22child*+teach*+close*%22+OR+%22child*+and+teach*+close*%22+OR+%22pupil*+teach*+close*%22+OR+%22pupil*+and+teach*+close*%22+OR+%22student*+teach*+attach*%22+OR+%22student*-teach*+attach*%22+OR+%22student*+and+teach*+attach*%22+OR+%22child*+teach*+attach*%22+OR+%22child*+and+teach*+attach*%22+OR+%22pupil*+teach*+attach*%22+OR+%22pupil*+and+teach*+attach*%22+OR+%22student*+teach*+warm*%22+OR+%22student*-teach*+warm*%22+OR+%22student*+and+teach*+warm*%22+OR+%22child*+teach*+warm*%22+OR+%22child*+and+teach*+warm*%22+OR+%22pupil*+teach*+warm*%22+OR+%22pupil*+and+teach*+warm*%22+OR+%22student*+teach*+support*%22+OR+%22student*-teach*+support*%22+OR+%22student*+and+teach*+support*%22+OR+%22child*+teach*+support*%22+OR+%22child*+and+teach*+support*%22+OR+%22pupil*+teach*+support*%22+OR+%22pupil*+and+teach*+support*%22+OR+%22student*+teach*+involv*%22+OR+%22student*-teach*+involv*%22+OR+%22student*+and+teach*+involv*%22+OR+%22child*+teach*+involv*%22+OR+%22child*+and+teach*+involv*%22+OR+%22pupil*+teach*+involv*%22+OR+%22pupil*+and+teach*+involv*%22+OR+%22student*+teach*+affiliat*%22+OR+%22student*-teach*+affiliat*%22+OR+%22student*+and+teach*+affiliat*%22+OR+%22child*+teach*+affiliat*%22+OR+%22child*+and+teach*+affiliat*%22+OR+%22pupil*+teach*+affiliat*%22+OR+%22pupil*+and+teach*+affiliat*%22+OR+%22student*+teach*+affect*%22+OR+%22student*-teach*+affect*%22+OR+%22student*+and+teach*+affect*%22+OR+%22child*+teach*+affect*%22+OR+%22child*+and+teach*+affect*%22+OR+%22pupil*+teach*+affect*%22+OR+%22pupil*+and+teach*+affect*%22+OR+%22student*+teach*+empat*%22+OR+%22student*-teach*+empat*%22+OR+%22student*+and+teach*+empat*%22+OR+%22child*+teach*+empat*%22+OR+%22child*+and+teach*+empat*%22+OR+%22pupil*+teach*+empat*%22+OR+%22pupil*+and+teach*+empat*%22+OR+%22student*+teach*+trust*%22+OR+%22student*-teach*+trust*%22+OR+%22student*+and+teach*+trust*%22+OR+%22child*+teach*+trust*%22+OR+%22child*+and+teach*+trust*%22+OR+%22pupil*+teach*+trust*%22+OR+%22pupil*+and+teach*+trust*%22+OR+%22student*+teach*+sensit*%22+OR+%22student*-teach*+sensit*%22+OR+%22student*+and+teach*+sensit*%22+OR+%22child*+teach*+sensit*%22+OR+%22child*+and+teach*+sensit*%22+OR+%22pupil*+teach*+sensit*%22+OR+%22pupil*+and+teach*+sensit*%22+OR+%22student*+teach*+respons*%22+OR+%22student*-teach*+respons*%22+OR+%22student*+and+teach*+respons*%22+OR+%22child*+teach*+respons*%22+OR+%22child*+and+teach*+respons*%22+OR+%22pupil*+teach*+respons*%22+OR+%22pupil*+and+teach*+respons*%22+OR+%22student*+teach*+lik*%22+OR+%22student*-teach*+lik*%22+OR+%22student*+and+teach*+lik*%22+OR+%22child*+teach*+lik*%22+OR+%22child*+and+teach*+lik*%22+OR+%22pupil*+teach*+lik*%22+OR+%22pupil*+and+teach*+lik*%22+OR+%22student*+teach*+dislik*%22+OR+%22student*-teach*+dislik*%22+OR+%22student*+and+teach*+dislik*%22+OR+%22child*+teach*+dislik*%22+OR+%22child*+and+teach*+dislik*%22+OR+%22pupil*+teach*+dislik*%22+OR+%22pupil*+and+teach*+dislik*%22+OR+%22student*+teach*+car*%22+OR+%22student*-teach*+car*%22+OR+%22student*+and+teach*+car*%22+OR+%22child*+teach*+car*%22+OR+%22child*+and+teach*+car*%22+OR+%22pupil*+teach*+car*%22+OR+%22pupil*+and+teach*+car*%22+OR+%22student*+teach*+conflict*%22+OR+%22student*-teach*+conflict*%22+OR+%22student*+and+teach*+conflict*%22+OR+%22child*+teach*+conflict*%22+OR+%22child*+and+teach*+conflict*%22+OR+%22pupil*+teach*+conflict*%22+OR+%22pupil*+and+teach*+conflict*%22+OR+%22student*+teach*+neglect*%22+OR+%22student*-teach*+neglect*%22+OR+%22student*+and+teach*+neglect*%22+OR+%22child*+teach*+neglect*%22+OR+%22child*+and+teach*+neglect*%22+OR+%22pupil*+teach*+neglect*%22+OR+%22pupil*+and+teach*+neglect*%22+OR+%22student*+teach*+reject*%22+OR+%22student*-teach*+reject*%22+OR+%22student*+and+teach*+reject*%22+OR+%22child*+teach*+reject*%22+OR+%22child*+and+teach*+reject*%22+OR+%22pupil*+teach*+reject*%22+OR+%22pupil*+and+teach*+reject*%22+OR+%22student*+teach*+negat*%22+OR+%22student*-teach*+negat*%22+OR+%22student*+and+teach*+negat*%22+OR+%22child*+teach*+negat*%22+OR+%22child*+and+teach*+negat*%22+OR+%22pupil*+teach*+negat*%22+OR+%22pupil*+and+teach*+negat*%22+OR+%22student*+teach*+ang*%22+OR+%22student*-teach*+ang*%22+OR+%22student*+and+teach*+ang*%22+OR+%22child*+teach*+ang*%22+OR+%22child*+and+teach*+ang*%22+OR+%22pupil*+teach*+ang*%22+OR+%22pupil*+and+teach*+ang*%22+OR+%22student*+teach*+concer*%22+OR+%22student*-teach*+concer*%22+OR+%22student*+and+teach*+concer*%22+OR+%22child*+teach*+concer*%22+OR+%22child*+and+teach*+concer*%22+OR+%22pupil*+teach*+concer*%22+OR+%22pupil*+and+teach*+concer*%22+OR+%22teach*+student*+rapport*%22+OR+%22teach*-student*+rapport*%22+OR+%22teach*+and+student*+rapport*%22+OR+%22teach*-child*+rapport*%22+OR+%22teach*+child*+rapport*%22+OR+%22teach*+and+child*+rapport*%22+OR+%22teach*-pupil*+rapport*%22+OR+%22teach*+pupil*+rapport*%22+OR+%22teach*+and+pupil*+rapport*%22+OR+%22student*+teach*+rapport*%22+OR+%22student*-teach*+rapport*%22+OR+%22student*+and+teach*+rapport*%22+OR+%22child*+teach*+rapport*%22+OR+%22child*+and+teach*+rapport*%22+OR+%22pupil*+teach*+rapport*%22+OR+%22pupil*+and+teach*+rapport*%22+OR+%22teach*+student*+encourage*%22+OR+%22teach*-student*+encourage*%22+OR+%22teach*+and+student*+encourage*%22+OR+%22teach*-child*+encourage*%22+OR+%22teach*+child*+encourage*%22+OR+%22teach*+and+child*+encourage*%22+OR+%22teach*-pupil*+encourage*%22+OR+%22teach*+pupil*+encourage*%22+OR+%22teach*+and+pupil*+encourage*%22+OR+%22student*+teach*+encourage*%22+OR+%22student*-teach*+encourage*%22+OR+%22student*+and+teach*+encourage*%22+OR+%22child*+teach*+encourage*%22+OR+%22child*+and+teach*+encourage*%22+OR+%22pupil*+teach*+encourage*%22+OR+%22pupil*+and+teach*+encourage*%22+OR+%22teach*+student*+engag*%22+OR+%22teach*-student*+engag*%22+OR+%22teach*+and+student*+engag*%22+OR+%22teach*-child*+engag*%22+OR+%22teach*+child*+engag*%22+OR+%22teach*+and+child*+engag*%22+OR+%22teach*-pupil*+engag*%22+OR+%22teach*+pupil*+engag*%22+OR+%22teach*+and+pupil*+engag*%22OR+%22student*+teach*+engag*%22+OR+%22student*-teach*+engag*%22+OR+%22student*+and+teach*+engag*%22+OR+%22child*+teach*+engag*%22+OR+%22child*+and+teach*+engag*%22+OR+%22pupil*+teach*+engag*%22+OR+%22pupil*+and+teach*+engag*%22+OR+%22teach*+student*+motiv*%22+OR+%22teach*-student*+motiv*%22+OR+%22teach*+and+student*+motiv*%22+OR+%22teach*-child*+motiv*%22+OR+%22teach*+child*+motiv*%22+OR+%22teach*+and+child*+motiv*%22+OR+%22teach*-pupil*+motiv*%22+OR+%22teach*+pupil*+motiv*%22+OR+%22teach*+and+pupil*+motiv*%22+OR+%22student*+teach*+motiv*%22+OR+%22student*-teach*+motiv*%22+OR+%22student*+and+teach*+motiv*%22+OR+%22child*+teach*+motiv*%22+OR+%22child*+and+teach*+motiv*%22+OR+%22pupil*+teach*+motiv*%22+OR+%22pupil*+and+teach*+motiv*%22+OR+%22teach*+student*+respect*%22+OR+%22teach*-student*+respect*%22+OR+%22teach*+and+student*+respect*%22+OR+%22teach*-child*+respect*%22+OR+%22teach*+child*+respect*%22+OR+%22teach*+and+child*+respect*%22+OR+%22teach*-pupil*+respect*%22+OR+%22teach*+pupil*+respect*%22+OR+%22teach*+and+pupil*+respect*%22+OR+%22student*+teach*+respect*%22+OR+%22student*-teach*+respect*%22+OR+%22student*+and+teach*+respect*%22+OR+%22child*+teach*+respect*%22+OR+%22child*+and+teach*+respect*%22+OR+%22pupil*+teach*+respect*%22+OR+%22pupil*+and+teach*+respect*%22+OR+%22teach*+student*+classroom+manag*%22+OR+%22teach*-student*+classroom+manag*%22+OR+%22teach*+and+student*+classroom+manag*%22+OR+%22teach*-child*+classroom+manag*%22+OR+%22teach*+child*+classroom+manag*%22+OR+%22teach*+and+child*+classroom+manag*%22+OR+%22teach*-pupil*+classroom+manag*%22+OR+%22teach*+pupil*+classroom+manag*%22+OR+%22teach*+and+pupil*+classroom+manag*%22+OR+%22student*+teach*+classroom+manag*%22+OR+%22student*-teach*+classroom+manag*%22+OR+%22student*+and+teach*+classroom+manag*%22+OR+%22child*+teach*+classroom+manag*%22+OR+%22child*+and+teach*+classroom+manag*%22+OR+%22pupil*+teach*+classroom+manag*%22+OR+%22pupil*+and+teach*+classroom+manag*%22+OR+%22teach*+student*+communicat*%22+OR+%22teach*-student*+communicat*%22+OR+%22teach*+and+student*+communicat*%22+OR+%22teach*-child*+communicat*%22+OR+%22teach*+child*+communicat*%22+OR+%22teach*+and+child*+communicat*%22+OR+%22teach*-pupil*+communicat*%22+OR+%22teach*+pupil*+communicat*%22+OR+%22teach*+and+pupil*+communicat*%22+OR+%22student*+teach*+communicat*%22+OR+%22student*-teach*+communicat*%22+OR+%22student*+and+teach*+communicat*%22+OR+%22child*+teach*+communicat*%22+OR+%22child*+and+teach*+communicat*%22+OR+%22pupil*+teach*+communicat*%22+OR+%22pupil*+and+teach*+communicat*%22+OR+%22teach*+student*+conflict*%22+OR+%22teach*-student*+conflict*%22+OR+%22teach*+and+student*+conflict*%22+OR+%22teach*-child*+conflict*%22+OR+%22teach*+child*+conflict*%22+OR+%22teach*+and+child*+conflict*%22+OR+%22teach*-pupil*+conflict*%22+OR+%22teach*+pupil*+conflict*%22+OR+%22teach*+and+pupil*+conflict*%22+OR+%22student*+teach*+conflict*%22+OR+%22student*-teach*+conflict*%22+OR+%22student*+and+teach*+conflict*%22+OR+%22child*+teach*+conflict*%22+OR+%22child*+and+teach*+conflict*%22+OR+%22pupil*+teach*+conflict*%22+OR+%22pupil*+and+teach*+conflict*%22+OR+%22teach*+student*+hostil*%22+OR+%22teach*-student*+hostil*%22+OR+%22teach*+and+student*+hostil*%22+OR+%22teach*-child*+hostil*%22+OR+%22teach*+child*+hostil*%22+OR+%22teach*+and+child*+hostil*%22+OR+%22teach*-pupil*+hostil*%22+OR+%22teach*+pupil*+hostil*%22+OR+%22teach*+and+pupil*+hostil*%22+OR+%22student*+teach*+hostil*%22+OR+%22student*-teach*+hostil*%22+OR+%22student*+and+teach*+hostil*%22+OR+%22child*+teach*+hostil*%22+OR+%22child*+and+teach*+hostil*%22+OR+%22pupil*+teach*+hostil*%22+OR+%22pupil*+and+teach*+hostil*%22+OR+%22teach*+student*+tens*%22+OR+%22teach*-student*+tens*%22+OR+%22teach*+and+student*+tens*%22+OR+%22teach*-child*+tens*%22+OR+%22teach*+child*+tens*%22+OR+%22teach*+and+child*+tens*%22+OR+%22teach*-pupil*+tens*%22+OR+%22teach*+pupil*+tens*%22+OR+%22teach*+and+pupil*+tens*%22+OR+%22student*+teach*+tens*%22+OR+%22student*-teach*+tens*%22+OR+%22student*+and+teach*+tens*%22+OR+%22child*+teach*+tens*%22+OR+%22child*+and+teach*+tens*%22+OR+%22pupil*+teach*+tens*%22+OR+%22pupil*+and+teach*+tens*%22+OR+%22teach*+student*+disagr*%22+OR+%22teach*-student*+disagr*%22+OR+%22teach*+and+student*+disagr*%22+OR+%22teach*-child*+disagr*%22+OR+%22teach*+child*+disagr*%22+OR+%22teach*+and+child*+disagr*%22+OR+%22teach*-pupil*+disagr*%22+OR+%22teach*+pupil*+disagr*%22+OR+%22teach*+and+pupil*+disagr*%22+OR+%22student*+teach*+disagr*%22+OR+%22student*-teach*+disagr*%22+OR+%22student*+and+teach*+disagr*%22+OR+%22child*+teach*+disagr*%22+OR+%22child*+and+teach*+disagr*%22+OR+%22pupil*+teach*+disagr*%22+OR+%22pupil*+and+teach*+disagr*%22+OR+%22teach*+student*+critic*%22+OR+%22teach*-student*+critic*%22+OR+%22teach*+and+student*+critic*%22+OR+%22teach*-child*+critic*%22+OR+%22teach*+child*+critic*%22+OR+%22teach*+and+child*+critic*%22+OR+%22teach*-pupil*+critic*%22+OR+%22teach*+pupil*+critic*%22+OR+%22teach*+and+pupil*+critic*%22+OR+%22student*+teach*+critic*%22+OR+%22student*-teach*+critic*%22+OR+%22student*+and+teach*+critic*%22+OR+%22child*+teach*+critic*%22+OR+%22child*+and+teach*+critic*%22+OR+%22pupil*+teach*+critic*%22+OR+%22pupil*+and+teach*+critic*%22%29&sid=edfda7a46d0e2d85d087e768801697db&sot=b&sdt=b&sessionSearchId=edfda7a46d0e2d85d087e768801697db&origin=searchbasic&editSaveSearch=&yearFrom=Before+1960&yearTo=Present&limit=10

- L2 + Population + Achievement Emotions + Working Memory: **3** results from Scopus

https://www.scopus.com/results/results.uri?sort=plf-f&src=s&st1=%28L2+OR+%22L2+learn*%22+OR+%22second+language*%22+OR+%22second+language+learning%22+or+%22foreign+language*%22+OR+FL+OR+%22Second+Language+Acquisition%22+OR+SLA%29+AND+%28%22primary+school*%22+or+%22elementary+school*%22+or+%22elementary+student*%22+or+%22primary+student*%22%29+AND+%28%22anxiet*%22+OR+%22sham*%22+OR+%22ang*%22+OR+%22enjo*%22+OR+%22bor*%22+OR+%22hop*%22+OR+%22prid*%22+OR+%22joy*%22+OR+%22frustrat*%22+OR+%22relie*%22+OR+%22relax*%22+OR+%22content*%22+OR+%22disapp*%22+OR+%22sad*%22+OR+%22grat*%22+OR+%22achievement+emot*%22+OR+%22academic+emot*%22+OR+%22emot*%22%29+AND+%28%22working+memor*%22+or+WM+or+%22immediate+memor*%22+or+%22operant+memor*%22+or+%22provisional+memor*%22+or+%22short-term+memor*%22+OR+STM+or+%22affective+working+memor*%22+or+%22emotional+working+memor*%22+or+%22working+memor*+for+valenc*+stimul*%22+or+%22working+memor*+for+emot*+stimul*%22%29&sid=a7db3c598008338a299cc3246a4c7a7b&sot=b&sdt=b&sl=743&s=TITLE-ABS-KEY%28%28L2+OR+%22L2+learn*%22+OR+%22second+language*%22+OR+%22second+language+learning%22+or+%22foreign+language*%22+OR+FL+OR+%22Second+Language+Acquisition%22+OR+SLA%29+AND+%28%22primary+school*%22+or+%22elementary+school*%22+or+%22elementary+student*%22+or+%22primary+student*%22%29+AND+%28%22anxiet*%22+OR+%22sham*%22+OR+%22ang*%22+OR+%22enjo*%22+OR+%22bor*%22+OR+%22hop*%22+OR+%22prid*%22+OR+%22joy*%22+OR+%22frustrat*%22+OR+%22relie*%22+OR+%22relax*%22+OR+%22content*%22+OR+%22disapp*%22+OR+%22sad*%22+OR+%22grat*%22+OR+%22achievement+emot*%22+OR+%22academic+emot*%22+OR+%22emot*%22%29+AND+%28%22working+memor*%22+or+WM+or+%22immediate+memor*%22+or+%22operant+memor*%22+or+%22provisional+memor*%22+or+%22short-term+memor*%22+OR+STM+or+%22affective+working+memor*%22+or+%22emotional+working+memor*%22+or+%22working+memor*+for+valenc*+stimul*%22+or+%22working+memor*+for+emot*+stimul*%22%29%29&origin=searchbasic&editSaveSearch=&yearFrom=Before+1960&yearTo=Present&sessionSearchId=a7db3c598008338a299cc3246a4c7a7b&limit=10

- L2 + **POPULATION** + ACHIEVEMENT EMOTIONS + STUDENT-TEACHER RELATIONSHIP:
- **1** result from Scopus
- https://www.scopus.com/results/results.uri?sort=plf-f&src=s&st1=%28L2+OR+%22L2+learn*%22+OR+%22second+language*%22+OR+%22second+language+learning%22+or+%22foreign+language*%22+OR+FL+OR+%22Second+Language+Acquisition%22+OR+SLA%29+AND+%28%22primary+school*%22+or+%22elementary+school*%22+or+%22elementary+student*%22+or+%22primary+student*%22%29+AND+%28%22anxiet*%22+OR+%22sham*%22+OR+%22ang*%22+OR+%22enjo*%22+OR+%22bor*%22+OR+%22hop*%22+OR+%22prid*%22+OR+%22joy*%22+OR+%22frustrat*%22+OR+%22relie*%22+OR+%22relax*%22+OR+%22content*%22+OR+%22disapp*%22+OR+%22sad*%22+OR+%22grat*%22+OR+%22achievement+emot*%22+OR+%22academic+emot*%22+OR+%22emot*%22%29+AND+%28%22teach*+student*+relat*%22+OR+%22teach*-student*+relat*%22+OR+%22teach*+and+student*+relat*%22+OR+%22teach*-child*+relat*%22+OR+%22teach*+child*+relat*%22+OR+%22teach*+and+child*+relat*%22+OR+%22teach*-pupil*+relat*%22+OR+%22teach*+pupil*+relat*%22+OR+%22teach*+and+pupil*+relat*%22+OR+%22teach*+student*+close*%22+OR+%22teach*-student*+close*%22+OR+%22teach*+and+student*+close*%22+OR+%22teach*-child*+close*%22+OR+%22teach*+child*+close*%22+OR+%22teach*+and+child*+close*%22+OR+%22teach*-pupil*+close*%22+OR+%22teach*+pupil*+close*%22+OR+%22teach*+and+pupil*+close*%22+OR+%22teach*+student*+attach*%22+OR+%22teach*-student*+attach*%22+OR+%22teach*+and+student*+attach*%22+OR+%22teach*-child*+attach*%22+OR+%22teach*+child*+attach*%22+OR+%22teach*+and+child*+attach*%22+OR+%22teach*-pupil*+attach*%22+OR+%22teach*+pupil*+attach*%22+OR+%22teach*+and+pupil*+attach*%22+OR+%22teach*+student*+warm*%22+OR+%22teach*-student*+warm*%22+OR+%22teach*+and+student*+warm*%22+OR+%22teach*-child*+warm*%22+OR+%22teach*+child*+warm*%22+OR+%22teach*+and+child*+warm*%22+OR+%22teach*-pupil*+warm*%22+OR+%22teach*+pupil*+warm*%22+OR+%22teach*+and+pupil*+warm*%22+OR+%22teach*+student*+support*%22+OR+%22teach*-student*+support*%22+OR+%22teach*+and+student*+support*%22+OR+%22teach*-child*+support*%22+OR+%22teach*+child*+support*%22+OR+%22teach*+and+child*+support*%22+OR+%22teach*-pupil*+support*%22+OR+%22teach*+pupil*+support*%22+OR+%22teach*+and+pupil*+support*%22+OR+%22teach*+student*+involv*%22+OR+%22teach*-student*+involv*%22+OR+%22teach*+and+student*+involv*%22+OR+%22teach*-child*+involv*%22+OR+%22teach*+child*+involv*%22+OR+%22teach*+and+child*+involv*%22+OR+%22teach*-pupil*+involv*%22+OR+%22teach*+pupil*+involv*%22+OR+%22teach*+and+pupil*+involv*%22+OR+%22teach*+student*+affiliat*%22+OR+%22teach*-student*+affiliat*%22+OR+%22teach*+and+student*+affiliat*%22+OR+%22teach*-child*+affiliat*%22+OR+%22teach*+child*+affiliat*%22+OR+%22teach*+and+child*+affiliat*%22+OR+%22teach*-pupil*+affiliat*%22+OR+%22teach*+pupil*+affiliat*%22+OR+%22teach*+and+pupil*+affiliat*%22+OR+%22teach*+student*+affect*%22+OR+%22teach*-student*+affect*%22+OR+%22teach*+and+student*+affect*%22+OR+%22teach*-child*+affect*%22+OR+%22teach*+child*+affect*%22+OR+%22teach*+and+child*+affect*%22+OR+%22teach*-pupil*+affect*%22+OR+%22teach*+pupil*+affect*%22+OR+%22teach*+and+pupil*+affect*%22+OR+%22teach*+student*+empat*%22+OR+%22teach*-student*+empat*%22+OR+%22teach*+and+student*+empat*%22+OR+%22teach*-child*+empat*%22+OR+%22teach*+child*+empat*%22+OR+%22teach*+and+child*+empat*%22+OR+%22teach*-pupil*+empat*%22+OR+%22teach*+pupil*+empat*%22+OR+%22teach*+and+pupil*+empat*%22+OR+%22teach*+student*+trust*%22+OR+%22teach*-student*+trust*%22+OR+%22teach*+and+student*+trust*%22+OR+%22teach*-child*+trust*%22+OR+%22teach*+child*+trust*%22+OR+%22teach*+and+child*+trust*%22+OR+%22teach*-pupil*+trust*%22+OR+%22teach*+pupil*+trust*%22+OR+%22teach*+and+pupil*+trust*%22+OR+%22teach*+student*+sensit*%22+OR+%22teach*-student*+sensit*%22+OR+%22teach*+and+student*+sensit*%22+OR+%22teach*-child*+sensit*%22+OR+%22teach*+child*+sensit*%22+OR+%22teach*+and+child*+sensit*%22+OR+%22teach*-pupil*+sensit*%22+OR+%22teach*+pupil*+sensit*%22+OR+%22teach*+and+pupil*+sensit*%22+OR+%22teach*+student*+respons*%22+OR+%22teach*-student*+respons*%22+OR+%22teach*+and+student*+respons*%22+OR+%22teach*-child*+respons*%22+OR+%22teach*+child*+respons*%22+OR+%22teach*+and+child*+respons*%22+OR+%22teach*-pupil*+respons*%22+OR+%22teach*+pupil*+respons*%22+OR+%22teach*+and+pupil*+respons*%22+OR+%22teach*+student*+lik*%22+OR+%22teach*-student*+lik*%22+OR+%22teach*+and+student*+lik*%22+OR+%22teach*-child*+lik*%22+OR+%22teach*+child*+lik*%22+OR+%22teach*+and+child*+lik*%22+OR+%22teach*-pupil*+lik*%22+OR+%22teach*+pupil*+lik*%22+OR+%22teach*+and+pupil*+lik*%22+OR+%22teach*+student*+dislik*%22+OR+%22teach*-student*+dislik*%22+OR+%22teach*+and+student*+dislik*%22+OR+%22teach*-child*+dislik*%22+OR+%22teach*+child*+dislik*%22+OR+%22teach*+and+child*+dislik*%22+OR+%22teach*-pupil*+dislik*%22+OR+%22teach*+pupil*+dislik*%22+OR+%22teach*+and+pupil*+dislik*%22+OR+%22teach*+student*+car*%22+OR+%22teach*-student*+car*%22+OR+%22teach*+and+student*+car*%22+OR+%22teach*-child*+car*%22+OR+%22teach*+child*+car*%22+OR+%22teach*+and+child*+car*%22+OR+%22teach*-pupil*+car*%22+OR+%22teach*+pupil*+car*%22+OR+%22teach*+and+pupil*+car*%22+OR+%22teach*+student*+conflict*%22+OR+%22teach*-student*+conflict*%22+OR+%22teach*+and+student*+conflict*%22+OR+%22teach*-child*+conflict*%22+OR+%22teach*+child*+conflict*%22+OR+%22teach*+and+child*+conflict*%22+OR+%22teach*-pupil*+conflict*%22+OR+%22teach*+pupil*+conflict*%22+OR+%22teach*+and+pupil*+conflict*%22+OR+%22teach*+student*+neglect*%22+OR+%22teach*-student*+neglect*%22+OR+%22teach*+and+student*+neglect*%22+OR+%22teach*-child*+neglect*%22+OR+%22teach*+child*+neglect*%22+OR+%22teach*+and+child*+neglect*%22+OR+%22teach*-pupil*+neglect*%22+OR+%22teach*+pupil*+neglect*%22+OR+%22teach*+and+pupil*+neglect*%22+OR+%22teach*+student*+reject*%22+OR+%22teach*-student*+reject*%22+OR+%22teach*+and+student*+reject*%22+OR+%22teach*-child*+reject*%22+OR+%22teach*+child*+reject*%22+OR+%22teach*+and+child*+reject*%22+OR+%22teach*-pupil*+reject*%22+OR+%22teach*+pupil*+reject*%22+OR+%22teach*+and+pupil*+reject*%22+OR+%22teach*+student*+negat*%22+OR+%22teach*-student*+negat*%22+OR+%22teach*+and+student*+negat*%22+OR+%22teach*-child*+negat*%22+OR+%22teach*+child*+negat*%22+OR+%22teach*+and+child*+negat*%22+OR+%22teach*-pupil*+negat*%22+OR+%22teach*+pupil*+negat*%22+OR+%22teach*+and+pupil*+negat*%22+OR+%22teach*+student*+ang*%22+OR+%22teach*-student*+ang*%22+OR+%22teach*+and+student*+ang*%22+OR+%22teach*-child*+ang*%22+OR+%22teach*+child*+ang*%22+OR+%22teach*+and+child*+ang*%22+OR+%22teach*-pupil*+ang*%22+OR+%22teach*+pupil*+ang*%22+OR+%22teach*+and+pupil*+ang*%22+OR+%22teach*+student*+concer*%22+OR+%22teach*-student*+concer*%22+OR+%22teach*+and+student*+concer*%22+OR+%22teach*-child*+concer*%22+OR+%22teach*+child*+concer*%22+OR+%22teach*+and+child*+concer*%22+OR+%22teach*-pupil*+concer*%22+OR+%22teach*+pupil*+concer*%22+OR+%22teach*+and+pupil*+concer*%22+OR+%22student*+teach*+relat*%22+OR+%22student*-teach*+relat*%22+OR+%22student*+and+teach*+relat*%22+OR+%22child*+teach*+relat*%22+OR+%22child*+and+teach*+relat*%22+OR+%22pupil*+teach*+relat*%22+OR+%22pupil*+and+teach*+relat*%22+OR+%22student*+teach*+close*%22+OR+%22student*-teach*+close*%22+OR+%22student*+and+teach*+close*%22+OR+%22child*+teach*+close*%22+OR+%22child*+and+teach*+close*%22+OR+%22pupil*+teach*+close*%22+OR+%22pupil*+and+teach*+close*%22+OR+%22student*+teach*+attach*%22+OR+%22student*-teach*+attach*%22+OR+%22student*+and+teach*+attach*%22+OR+%22child*+teach*+attach*%22+OR+%22child*+and+teach*+attach*%22+OR+%22pupil*+teach*+attach*%22+OR+%22pupil*+and+teach*+attach*%22+OR+%22student*+teach*+warm*%22+OR+%22student*-teach*+warm*%22+OR+%22student*+and+teach*+warm*%22+OR+%22child*+teach*+warm*%22+OR+%22child*+and+teach*+warm*%22+OR+%22pupil*+teach*+warm*%22+OR+%22pupil*+and+teach*+warm*%22+OR+%22student*+teach*+support*%22+OR+%22student*-teach*+support*%22+OR+%22student*+and+teach*+support*%22+OR+%22child*+teach*+support*%22+OR+%22child*+and+teach*+support*%22+OR+%22pupil*+teach*+support*%22+OR+%22pupil*+and+teach*+support*%22+OR+%22student*+teach*+involv*%22+OR+%22student*-teach*+involv*%22+OR+%22student*+and+teach*+involv*%22+OR+%22child*+teach*+involv*%22+OR+%22child*+and+teach*+involv*%22+OR+%22pupil*+teach*+involv*%22+OR+%22pupil*+and+teach*+involv*%22+OR+%22student*+teach*+affiliat*%22+OR+%22student*-teach*+affiliat*%22+OR+%22student*+and+teach*+affiliat*%22+OR+%22child*+teach*+affiliat*%22+OR+%22child*+and+teach*+affiliat*%22+OR+%22pupil*+teach*+affiliat*%22+OR+%22pupil*+and+teach*+affiliat*%22+OR+%22student*+teach*+affect*%22+OR+%22student*-teach*+affect*%22+OR+%22student*+and+teach*+affect*%22+OR+%22child*+teach*+affect*%22+OR+%22child*+and+teach*+affect*%22+OR+%22pupil*+teach*+affect*%22+OR+%22pupil*+and+teach*+affect*%22+OR+%22student*+teach*+empat*%22+OR+%22student*-teach*+empat*%22+OR+%22student*+and+teach*+empat*%22+OR+%22child*+teach*+empat*%22+OR+%22child*+and+teach*+empat*%22+OR+%22pupil*+teach*+empat*%22+OR+%22pupil*+and+teach*+empat*%22+OR+%22student*+teach*+trust*%22+OR+%22student*-teach*+trust*%22+OR+%22student*+and+teach*+trust*%22+OR+%22child*+teach*+trust*%22+OR+%22child*+and+teach*+trust*%22+OR+%22pupil*+teach*+trust*%22+OR+%22pupil*+and+teach*+trust*%22+OR+%22student*+teach*+sensit*%22+OR+%22student*-teach*+sensit*%22+OR+%22student*+and+teach*+sensit*%22+OR+%22child*+teach*+sensit*%22+OR+%22child*+and+teach*+sensit*%22+OR+%22pupil*+teach*+sensit*%22+OR+%22pupil*+and+teach*+sensit*%22+OR+%22student*+teach*+respons*%22+OR+%22student*-teach*+respons*%22+OR+%22student*+and+teach*+respons*%22+OR+%22child*+teach*+respons*%22+OR+%22child*+and+teach*+respons*%22+OR+%22pupil*+teach*+respons*%22+OR+%22pupil*+and+teach*+respons*%22+OR+%22student*+teach*+lik*%22+OR+%22student*-teach*+lik*%22+OR+%22student*+and+teach*+lik*%22+OR+%22child*+teach*+lik*%22+OR+%22child*+and+teach*+lik*%22+OR+%22pupil*+teach*+lik*%22+OR+%22pupil*+and+teach*+lik*%22+OR+%22student*+teach*+dislik*%22+OR+%22student*-teach*+dislik*%22+OR+%22student*+and+teach*+dislik*%22+OR+%22child*+teach*+dislik*%22+OR+%22child*+and+teach*+dislik*%22+OR+%22pupil*+teach*+dislik*%22+OR+%22pupil*+and+teach*+dislik*%22+OR+%22student*+teach*+car*%22+OR+%22student*-teach*+car*%22+OR+%22student*+and+teach*+car*%22+OR+%22child*+teach*+car*%22+OR+%22child*+and+teach*+car*%22+OR+%22pupil*+teach*+car*%22+OR+%22pupil*+and+teach*+car*%22+OR+%22student*+teach*+conflict*%22+OR+%22student*-teach*+conflict*%22+OR+%22student*+and+teach*+conflict*%22+OR+%22child*+teach*+conflict*%22+OR+%22child*+and+teach*+conflict*%22+OR+%22pupil*+teach*+conflict*%22+OR+%22pupil*+and+teach*+conflict*%22+OR+%22student*+teach*+neglect*%22+OR+%22student*-teach*+neglect*%22+OR+%22student*+and+teach*+neglect*%22+OR+%22child*+teach*+neglect*%22+OR+%22child*+and+teach*+neglect*%22+OR+%22pupil*+teach*+neglect*%22+OR+%22pupil*+and+teach*+neglect*%22+OR+%22student*+teach*+reject*%22+OR+%22student*-teach*+reject*%22+OR+%22student*+and+teach*+reject*%22+OR+%22child*+teach*+reject*%22+OR+%22child*+and+teach*+reject*%22+OR+%22pupil*+teach*+reject*%22+OR+%22pupil*+and+teach*+reject*%22+OR+%22student*+teach*+negat*%22+OR+%22student*-teach*+negat*%22+OR+%22student*+and+teach*+negat*%22+OR+%22child*+teach*+negat*%22+OR+%22child*+and+teach*+negat*%22+OR+%22pupil*+teach*+negat*%22+OR+%22pupil*+and+teach*+negat*%22+OR+%22student*+teach*+ang*%22+OR+%22student*-teach*+ang*%22+OR+%22student*+and+teach*+ang*%22+OR+%22child*+teach*+ang*%22+OR+%22child*+and+teach*+ang*%22+OR+%22pupil*+teach*+ang*%22+OR+%22pupil*+and+teach*+ang*%22+OR+%22student*+teach*+concer*%22+OR+%22student*-teach*+concer*%22+OR+%22student*+and+teach*+concer*%22+OR+%22child*+teach*+concer*%22+OR+%22child*+and+teach*+concer*%22+OR+%22pupil*+teach*+concer*%22+OR+%22pupil*+and+teach*+concer*%22+OR+%22teach*+student*+rapport*%22+OR+%22teach*-student*+rapport*%22+OR+%22teach*+and+student*+rapport*%22+OR+%22teach*-child*+rapport*%22+OR+%22teach*+child*+rapport*%22+OR+%22teach*+and+child*+rapport*%22+OR+%22teach*-pupil*+rapport*%22+OR+%22teach*+pupil*+rapport*%22+OR+%22teach*+and+pupil*+rapport*%22+OR+%22student*+teach*+rapport*%22+OR+%22student*-teach*+rapport*%22+OR+%22student*+and+teach*+rapport*%22+OR+%22child*+teach*+rapport*%22+OR+%22child*+and+teach*+rapport*%22+OR+%22pupil*+teach*+rapport*%22+OR+%22pupil*+and+teach*+rapport*%22+OR+%22teach*+student*+encourage*%22+OR+%22teach*-student*+encourage*%22+OR+%22teach*+and+student*+encourage*%22+OR+%22teach*-child*+encourage*%22+OR+%22teach*+child*+encourage*%22+OR+%22teach*+and+child*+encourage*%22+OR+%22teach*-pupil*+encourage*%22+OR+%22teach*+pupil*+encourage*%22+OR+%22teach*+and+pupil*+encourage*%22+OR+%22student*+teach*+encourage*%22+OR+%22student*-teach*+encourage*%22+OR+%22student*+and+teach*+encourage*%22+OR+%22child*+teach*+encourage*%22+OR+%22child*+and+teach*+encourage*%22+OR+%22pupil*+teach*+encourage*%22+OR+%22pupil*+and+teach*+encourage*%22+OR+%22teach*+student*+engag*%22+OR+%22teach*-student*+engag*%22+OR+%22teach*+and+student*+engag*%22+OR+%22teach*-child*+engag*%22+OR+%22teach*+child*+engag*%22+OR+%22teach*+and+child*+engag*%22+OR+%22teach*-pupil*+engag*%22+OR+%22teach*+pupil*+engag*%22+OR+%22teach*+and+pupil*+engag*%22OR+%22student*+teach*+engag*%22+OR+%22student*-teach*+engag*%22+OR+%22student*+and+teach*+engag*%22+OR+%22child*+teach*+engag*%22+OR+%22child*+and+teach*+engag*%22+OR+%22pupil*+teach*+engag*%22+OR+%22pupil*+and+teach*+engag*%22+OR+%22teach*+student*+motiv*%22+OR+%22teach*-student*+motiv*%22+OR+%22teach*+and+student*+motiv*%22+OR+%22teach*-child*+motiv*%22+OR+%22teach*+child*+motiv*%22+OR+%22teach*+and+child*+motiv*%22+OR+%22teach*-pupil*+motiv*%22+OR+%22teach*+pupil*+motiv*%22+OR+%22teach*+and+pupil*+motiv*%22+OR+%22student*+teach*+motiv*%22+OR+%22student*-teach*+motiv*%22+OR+%22student*+and+teach*+motiv*%22+OR+%22child*+teach*+motiv*%22+OR+%22child*+and+teach*+motiv*%22+OR+%22pupil*+teach*+motiv*%22+OR+%22pupil*+and+teach*+motiv*%22+OR+%22teach*+student*+respect*%22+OR+%22teach*-student*+respect*%22+OR+%22teach*+and+student*+respect*%22+OR+%22teach*-child*+respect*%22+OR+%22teach*+child*+respect*%22+OR+%22teach*+and+child*+respect*%22+OR+%22teach*-pupil*+respect*%22+OR+%22teach*+pupil*+respect*%22+OR+%22teach*+and+pupil*+respect*%22+OR+%22student*+teach*+respect*%22+OR+%22student*-teach*+respect*%22+OR+%22student*+and+teach*+respect*%22+OR+%22child*+teach*+respect*%22+OR+%22child*+and+teach*+respect*%22+OR+%22pupil*+teach*+respect*%22+OR+%22pupil*+and+teach*+respect*%22+OR+%22teach*+student*+classroom+manag*%22+OR+%22teach*-student*+classroom+manag*%22+OR+%22teach*+and+student*+classroom+manag*%22+OR+%22teach*-child*+classroom+manag*%22+OR+%22teach*+child*+classroom+manag*%22+OR+%22teach*+and+child*+classroom+manag*%22+OR+%22teach*-pupil*+classroom+manag*%22+OR+%22teach*+pupil*+classroom+manag*%22+OR+%22teach*+and+pupil*+classroom+manag*%22+OR+%22student*+teach*+classroom+manag*%22+OR+%22student*-teach*+classroom+manag*%22+OR+%22student*+and+teach*+classroom+manag*%22+OR+%22child*+teach*+classroom+manag*%22+OR+%22child*+and+teach*+classroom+manag*%22+OR+%22pupil*+teach*+classroom+manag*%22+OR+%22pupil*+and+teach*+classroom+manag*%22+OR+%22teach*+student*+communicat*%22+OR+%22teach*-student*+communicat*%22+OR+%22teach*+and+student*+communicat*%22+OR+%22teach*-child*+communicat*%22+OR+%22teach*+child*+communicat*%22+OR+%22teach*+and+child*+communicat*%22+OR+%22teach*-pupil*+communicat*%22+OR+%22teach*+pupil*+communicat*%22+OR+%22teach*+and+pupil*+communicat*%22+OR+%22student*+teach*+communicat*%22+OR+%22student*-teach*+communicat*%22+OR+%22student*+and+teach*+communicat*%22+OR+%22child*+teach*+communicat*%22+OR+%22child*+and+teach*+communicat*%22+OR+%22pupil*+teach*+communicat*%22+OR+%22pupil*+and+teach*+communicat*%22+OR+%22teach*+student*+conflict*%22+OR+%22teach*-student*+conflict*%22+OR+%22teach*+and+student*+conflict*%22+OR+%22teach*-child*+conflict*%22+OR+%22teach*+child*+conflict*%22+OR+%22teach*+and+child*+conflict*%22+OR+%22teach*-pupil*+conflict*%22+OR+%22teach*+pupil*+conflict*%22+OR+%22teach*+and+pupil*+conflict*%22+OR+%22student*+teach*+conflict*%22+OR+%22student*-teach*+conflict*%22+OR+%22student*+and+teach*+conflict*%22+OR+%22child*+teach*+conflict*%22+OR+%22child*+and+teach*+conflict*%22+OR+%22pupil*+teach*+conflict*%22+OR+%22pupil*+and+teach*+conflict*%22+OR+%22teach*+student*+hostil*%22+OR+%22teach*-student*+hostil*%22+OR+%22teach*+and+student*+hostil*%22+OR+%22teach*-child*+hostil*%22+OR+%22teach*+child*+hostil*%22+OR+%22teach*+and+child*+hostil*%22+OR+%22teach*-pupil*+hostil*%22+OR+%22teach*+pupil*+hostil*%22+OR+%22teach*+and+pupil*+hostil*%22+OR+%22student*+teach*+hostil*%22+OR+%22student*-teach*+hostil*%22+OR+%22student*+and+teach*+hostil*%22+OR+%22child*+teach*+hostil*%22+OR+%22child*+and+teach*+hostil*%22+OR+%22pupil*+teach*+hostil*%22+OR+%22pupil*+and+teach*+hostil*%22+OR+%22teach*+student*+tens*%22+OR+%22teach*-student*+tens*%22+OR+%22teach*+and+student*+tens*%22+OR+%22teach*-child*+tens*%22+OR+%22teach*+child*+tens*%22+OR+%22teach*+and+child*+tens*%22+OR+%22teach*-pupil*+tens*%22+OR+%22teach*+pupil*+tens*%22+OR+%22teach*+and+pupil*+tens*%22+OR+%22student*+teach*+tens*%22+OR+%22student*-teach*+tens*%22+OR+%22student*+and+teach*+tens*%22+OR+%22child*+teach*+tens*%22+OR+%22child*+and+teach*+tens*%22+OR+%22pupil*+teach*+tens*%22+OR+%22pupil*+and+teach*+tens*%22+OR+%22teach*+student*+disagr*%22+OR+%22teach*-student*+disagr*%22+OR+%22teach*+and+student*+disagr*%22+OR+%22teach*-child*+disagr*%22+OR+%22teach*+child*+disagr*%22+OR+%22teach*+and+child*+disagr*%22+OR+%22teach*-pupil*+disagr*%22+OR+%22teach*+pupil*+disagr*%22+OR+%22teach*+and+pupil*+disagr*%22+OR+%22student*+teach*+disagr*%22+OR+%22student*-teach*+disagr*%22+OR+%22student*+and+teach*+disagr*%22+OR+%22child*+teach*+disagr*%22+OR+%22child*+and+teach*+disagr*%22+OR+%22pupil*+teach*+disagr*%22+OR+%22pupil*+and+teach*+disagr*%22+OR+%22teach*+student*+critic*%22+OR+%22teach*-student*+critic*%22+OR+%22teach*+and+student*+critic*%22+OR+%22teach*-child*+critic*%22+OR+%22teach*+child*+critic*%22+OR+%22teach*+and+child*+critic*%22+OR+%22teach*-pupil*+critic*%22+OR+%22teach*+pupil*+critic*%22+OR+%22teach*+and+pupil*+critic*%22+OR+%22student*+teach*+critic*%22+OR+%22student*-teach*+critic*%22+OR+%22student*+and+teach*+critic*%22+OR+%22child*+teach*+critic*%22+OR+%22child*+and+teach*+critic*%22+OR+%22pupil*+teach*+critic*%22+OR+%22pupil*+and+teach*+critic*%22%29&sid=bc73b604aee483c0ba81ac499014ffd3&sot=b&sdt=b&sessionSearchId=bc73b604aee483c0ba81ac499014ffd3&origin=searchbasic&editSaveSearch=&yearFrom=Before+1960&yearTo=Present&limit=10
- L2 + Population + Working Memory + Student-Teacher Relationship: 0 results
- L2 + Population + Achievement Emotions + Working Memory + Student-Teacher Relationship: 0 results

**Pubmed:**

- L2 + Population + Achievement Emotions: **69** results from Pubmed

<https://pubmed.ncbi.nlm.nih.gov/?term=%28L2+OR+%E2%80%9CL2+learn*%E2%80%9D+OR+%E2%80%9Csecond+language*%E2%80%9D+OR+%E2%80%9Csecond+language+learning%E2%80%9D+or+%E2%80%9Cforeign+language*%E2%80%9D+OR+FL+OR+%E2%80%9CSecond+Language+Acquisition%E2%80%9D+OR+SLA%29+AND+%28%E2%80%9Cprimary+school*%E2%80%9D+or+%E2%80%9Celementary+school*%E2%80%9D+or+%E2%80%9Celementary+student*%E2%80%9D+or+%E2%80%9Cprimary+student*%E2%80%9D%29+AND+%28%E2%80%9Canxiet*%E2%80%9D+OR+%E2%80%9Csham*%E2%80%9D+OR+%E2%80%9Cang*%E2%80%9D+OR+%E2%80%9Cenjo*%E2%80%9D+OR+%E2%80%9Cbor*%E2%80%9D+OR+%E2%80%9Chop*%E2%80%9D+OR+%E2%80%9Cprid*%E2%80%9D+OR+%E2%80%9Cjoy*%E2%80%9D+OR+%E2%80%9Cfrustrat*%E2%80%9D+OR+%E2%80%9Crelie*%E2%80%9D+OR+%E2%80%9Crelax*%E2%80%9D+OR+%E2%80%9Ccontent*%E2%80%9D+OR+%E2%80%9Cdisapp*%E2%80%9D+OR+%E2%80%9Csad*%E2%80%9D+OR+%E2%80%9Cgrat*%E2%80%9D+OR+%E2%80%9Cachievement+emot*%E2%80%9D+OR+%E2%80%9Cacademic+emot*%E2%80%9D+OR+%E2%80%9Cemot*%E2%80%9D%29&sort=>

- L2 + Population + Working Memory: **15** results from Pubmed

<https://pubmed.ncbi.nlm.nih.gov/?term=%28L2+OR+%E2%80%9CL2+learn*%E2%80%9D+OR+%E2%80%9Csecond+language*%E2%80%9D+OR+%E2%80%9Csecond+language+learning%E2%80%9D+or+%E2%80%9Cforeign+language*%E2%80%9D+OR+FL+OR+%E2%80%9CSecond+Language+Acquisition%E2%80%9D+OR+SLA%29+AND+%28%E2%80%9Cprimary+school*%E2%80%9D+or+%E2%80%9Celementary+school*%E2%80%9D+or+%E2%80%9Celementary+student*%E2%80%9D+or+%E2%80%9Cprimary+student*%E2%80%9D%29+AND+%28%E2%80%9Cworking+memor*%E2%80%9D+or+WM+or+%E2%80%9Cimmediate+memor*%E2%80%9D+or+%E2%80%9Coperant+memor*%E2%80%9D+or+%E2%80%9Cprovisional+memor*%E2%80%9D+or+%E2%80%9Cshort-term+memor*%E2%80%9D+OR+STM+or+%E2%80%9Caffective+working+memor*%E2%80%9D+or+%E2%80%9Cemotional+working+memor*%E2%80%9D+or+%E2%80%9Cworking+memor*+for+valenc*+stimul*%E2%80%9D+or+%E2%80%9Cworking+memor*+for+emot*+stimul*%E2%80%9D%29>

- L2 + Population + Student-Teacher Relationship: **0** results from Pubmed
- L2 + Population + Achievement Emotions + Working Memory: **2** results from Pubmed

https://pubmed.ncbi.nlm.nih.gov/?term=%28L2+OR+%E2%80%9CL2+learn*%E2%80%9D+OR+%E2%80%9Csecond+language*%E2%80%9D+OR+%E2%80%9Csecond+language+learning%E2%80%9D+or+%E2%80%9Cforeign+language*%E2%80%9D+OR+FL+OR+%E2%80%9CSecond+Language+Acquisition%E2%80%9D+OR+SLA%29+AND+%28%E2%80%9Cprimary+school*%E2%80%9D+or+%E2%80%9Celementary+school*%E2%80%9D+or+%E2%80%9Celementary+student*%E2%80%9D+or+%E2%80%9Cprimary+student*%E2%80%9D%29+AND+%28%E2%80%9Canxiet*%E2%80%9D+OR+%E2%80%9Csham*%E2%80%9D+OR+%E2%80%9Cang*%E2%80%9D+OR+%E2%80%9Cenjo*%E2%80%9D+OR+%E2%80%9Cbor*%E2%80%9D+OR+%E2%80%9Chop*%E2%80%9D+OR+%E2%80%9Cprid*%E2%80%9D+OR+%E2%80%9Cjoy*%E2%80%9D+OR+%E2%80%9Cfrustrat*%E2%80%9D+OR+%E2%80%9Crelie*%E2%80%9D+OR+%E2%80%9Crelax*%E2%80%9D+OR+%E2%80%9Ccontent*%E2%80%9D+OR+%E2%80%9Cdisapp*%E2%80%9D+OR+%E2%80%9Csad*%E2%80%9D+OR+%E2%80%9Cgrat*%E2%80%9D+OR+%E2%80%9Cachievement+emot*%E2%80%9D+OR+%E2%80%9Cacademic+emot*%E2%80%9D+OR+%E2%80%9Cemot*%E2%80%9D%29+AND+%28%E2%80%9Cworking+memor*%E2%80%9D+or+WM+or+%E2%80%9Cimmediate+memor*%E2%80%9D+or+%E2%80%9Coperant+memor*%E2%80%9D+or+%E2%80%9Cprovisional+memor*%E2%80%9D+or+%E2%80%9Cshort-term+memor*%E2%80%9D+OR+STM+or+%E2%80%9Caffective+working+memor*%E2%80%9D+or+%E2%80%9Cemotional+working+memor*%E2%80%9D+or+%E2%80%9Cworking+memor*+for+valenc*+stimul*%E2%80%9D+or+%E2%80%9Cworking+memor*+for+emot*+stimul*%E2%80%9D%29

- L2 + Population + Achievement Emotions + Student-Teacher Relationship:**0** result from Pubmed
- L2 + Population + Working Memory + Student-Teacher Relationship: **0** result from Pubmed
- L2 + Population + Achievement Emotions + Working Memory + Student-Teacher Relationship: **0** result from Pubmed

**APA PsycArticles :**

- L2 + Population + Achievement Emotions: **31** results <https://web.p.ebscohost.com/ehost/resultsadvanced?vid=5&sid=9dc9b3de-a1bc-4152-b4f0-7de4f17bda71%40redis&bquery=(L2+OR+%e2%80%9cL2+learn*%e2%80%9d+OR+%e2%80%9csecond+language*%e2%80%9d+OR+%e2%80%9csecond+language+learning%e2%80%9d+or+%e2%80%9cforeign+language*%e2%80%9d+OR+FL+OR+%e2%80%9cSecond+Language+Acquisition%e2%80%9d+OR+SLA)+AND+(%e2%80%9cprimary+school*%e2%80%9d+or+%e2%80%9celementary+school*%e2%80%9d+or+%e2%80%9celementary+student*%e2%80%9d+or+%e2%80%9cprimary+student*%e2%80%9d)+AND+(%e2%80%9canxiet*%e2%80%9d+OR+%e2%80%9csham*%e2%80%9d+OR+%e2%80%9cang*%e2%80%9d+OR+%e2%80%9cenjo*%e2%80%9d+OR+%e2%80%9cbor*%e2%80%9d+OR+%e2%80%9chop*%e2%80%9d+OR+%e2%80%9cprid*%e2%80%9d+OR+%e2%80%9cjoy*%e2%80%9d+OR+%e2%80%9cfrustrat*%e2%80%9d+OR+%e2%80%9crelie*%e2%80%9d+OR+%e2%80%9crelax*%e2%80%9d+OR+%e2%80%9ccontent*%e2%80%9d+OR+%e2%80%9cdisapp*%e2%80%9d+OR+%e2%80%9csad*%e2%80%9d+OR+%e2%80%9cgrat*%e2%80%9d+OR+%e2%80%9cachievement+emot*%e2%80%9d+OR+%e2%80%9cacademic+emot*%e2%80%9d+OR+%e2%80%9cemot*%e2%80%9d)&bdata=JmRiPXBkaCZ0eXBlPTEmc2VhcmNoTW9kZT1TdGFuZGFyZCZzaXRlPWVob3N0LWxpdmU%3d>
- L2 + Population + Working Memory: **9** results

<https://web.p.ebscohost.com/ehost/resultsadvanced?vid=6&sid=9dc9b3de-a1bc-4152-b4f0-7de4f17bda71%40redis&bquery=(L2+OR+%e2%80%9cL2+learn*%e2%80%9d+OR+%e2%80%9csecond+language*%e2%80%9d+OR+%e2%80%9csecond+language+learning%e2%80%9d+or+%e2%80%9cforeign+language*%e2%80%9d+OR+FL+OR+%e2%80%9cSecond+Language+Acquisition%e2%80%9d+OR+SLA)+AND+(%e2%80%9cprimary+school*%e2%80%9d+or+%e2%80%9celementary+school*%e2%80%9d+or+%e2%80%9celementary+student*%e2%80%9d+or+%e2%80%9cprimary+student*%e2%80%9d)+AND+(%e2%80%9cworking+memor*%e2%80%9d+or+WM+or+%e2%80%9cimmediate+memor*%e2%80%9d+or+%e2%80%9coperant+memor*%e2%80%9d+or+%e2%80%9cprovisional+memor*%e2%80%9d+or+%e2%80%9cshort-term+memor*%e2%80%9d+OR+STM+or+%e2%80%9caffective+working+memor*%e2%80%9d+or+%e2%80%9cemotional+working+memor*%e2%80%9d+or+%e2%80%9cworking+memor*+for+valenc*+stimul*%e2%80%9d+or+%e2%80%9cworking+memor*+for+emot*+stimul*%e2%80%9d)&bdata=JmRiPXBkaCZ0eXBlPTEmc2VhcmNoTW9kZT1TdGFuZGFyZCZzaXRlPWVob3N0LWxpdmU%3d>

- L2 + Population + Student-Teacher Relationship: **0** results
- L2 + Population + Achievement Emotions + Working Memory: **0** results
- L2 + Population + Achievement Emotions + Student-Teacher Relationship: **0** results
- L2 + Population + Working Memory + Student-Teacher Relationship: **0** results
- L2 + Population + Achievement Emotions + Working Memory + Student-Teacher Relationship: **0** results
