# Supplementary material for: A systematic review and meta-analysis on achievement emotions, working memory and student-teacher relationship during second language learning in primary school
Source: PLoS One. 2026 May 26;21(5):e0350119. doi: 10.1371/journal.pone.0350119 (PMC13210231; doi:10.1371/journal.pone.0350119)
Supplement: S2 File — (DOCX) [file pone.0350119.s002.docx]

**Supporting information for**

***A systematic review and metanalysis on achievement emotions, working memory and student-teacher relationship during L2 learning in primary school.***

1. **Additional information on quantitative analyses.**
   1. **Step-by-step: conversions to Pearson’s r**
      1. **Working memory**

- Aadland et al. (2017):

$$r_{Average}= \frac{.28+ .30}{2}= .29$$

- Chan et al. (2024):

$$r_{Average}= \frac{.14+ .06}{2}= .10$$

- Gillet et al. (2020):

$$r=\sqrt{{\eta p}^{2}}=\sqrt{.10}\approx.316\approx.32$$

- Kersten (2022):

$$\beta=0.42\times0.21=0.0882\approx0.09$$

$$\beta=r\approx0.09$$

- Mingjia and Xian (2025):

$$r_{Average}= \frac{.37+ .42+.41+20}{4}= .35$$

- Trotta et al. (2026):

$$r_{Average}= \frac{.13+.15+.18+.16+.09+.16+.26+.24+.25}{9}= .18$$

- - 1. **Achievement emotions**
- Chan et al. (2024):

$$r_{Average}= \frac{-.47+(-.46)}{2}= -.465= -.47$$

- Chen (2025):

| **Group** | **N** | **M** | **SD** |
| --- | --- | --- | --- |
| Low anxiety | 15 | 17.80 | 3.03 |
| High anxiety | 15 | 23.40 | 7.48 |

- ${SD}_{pooled}= \sqrt{\frac{\left( n_{1}-1 \right)s_{1}^{2}+(n_{2}-1)s_{2}^{2}}{{n_{1}+n}_{2}-2}}=\sqrt{\frac{\left( 15-1 \right){3.03}^{2}+(15-1){7.48}^{2}}{15+15-2}}=\sqrt{\frac{128.17+783.92}{28}= \sqrt{32.57}}\approx5.71$
- $d=\frac{M_{2}- M_{1}}{{SD}_{pooled}}= \frac{23.40-17.80}{5.71}\approx\frac{5.6}{5.71}\approx0.980$
- $J=1-\frac{3}{4\left( n_{1}+n_{2} \right)-9} \Rightarrow g=d\cdot J$
- $J=1-\frac{3}{4\left( 30 \right)-9}=1-\frac{3}{111}\approx0.973 \Rightarrow g=0.980\cdot0.973\approx0.955$
- $r=\frac{g}{\sqrt{g^{2}+4}}=\frac{0.955}{\sqrt{{0.955}^{2}+4}}=\frac{0.955}{\sqrt{0.912+4}}=\frac{0.955}{\sqrt{4.912}}\approx\frac{0.955}{2.216}\approx0.431$
- Liu and Hong (2025):

| **Grade** | **N** | **ESAS (Anxiety)** | | **ELCES (Enjoyment)** | |
| --- | --- | --- | --- | --- | --- |
|  |  | **M** | **SD** | **M** | **SD** |
| 4 | 144 | 2.30 | 0.79 | 3.39 | 0.696 |
| 5 | 123 | 2.51 | 0.84 | 3.25 | 0.60 |

1. ESAS (Anxiety)

$${SD}_{pooled}= \sqrt{\frac{\left( n_{1}-1 \right)s_{1}^{2}+(n_{2}-1)s_{2}^{2}}{{n_{1}+n}_{2}-2}}=\sqrt{\frac{\left( 144-1 \right){0.79}^{2}+(123-1){0.84}^{2}}{144+123-2}}=0.815$$

$$r=\frac{M_{2}- M_{1}}{{SD}_{pooled}}\cdot\sqrt{\frac{n_{1}\cdot n_{2}}{{{(n}_{1}+n_{2})}^{2}}}=\frac{2.30-2.51}{0.652}\cdot\sqrt{\frac{144\cdot123}{\left( 144+123 \right)^{2}}}=-.21$$

1. ELCES (Enjoyment)

$${SD}_{pooled}=0.652$$

$$r=\frac{3.39-3.25}{0.652}\cdot\sqrt{\frac{144\cdot123}{\left( 144+123 \right)^{2}}}=.15$$

- Trotta et al. (2026):

| Emotion | T1 | T2 | T3 | T4 | Mean_r |
| --- | --- | --- | --- | --- | --- |
| Enjoyment | 0.08 | 0.07 | 0.05 | 0.20 | 0.10 |
| Boredom | -0.09 | -0.1 | -0.12 | -0.1 | -0.10 |
| Anxiety | -0.16 | -0.13 | -0.22 | -0.02 | -0.13 |

- 1. **Sensitivity analyses**
     1. **Working memory and L2 learning**

**Figure S1.**

Diagnostic graphs of the influence of studies on working memory and L2 learning.


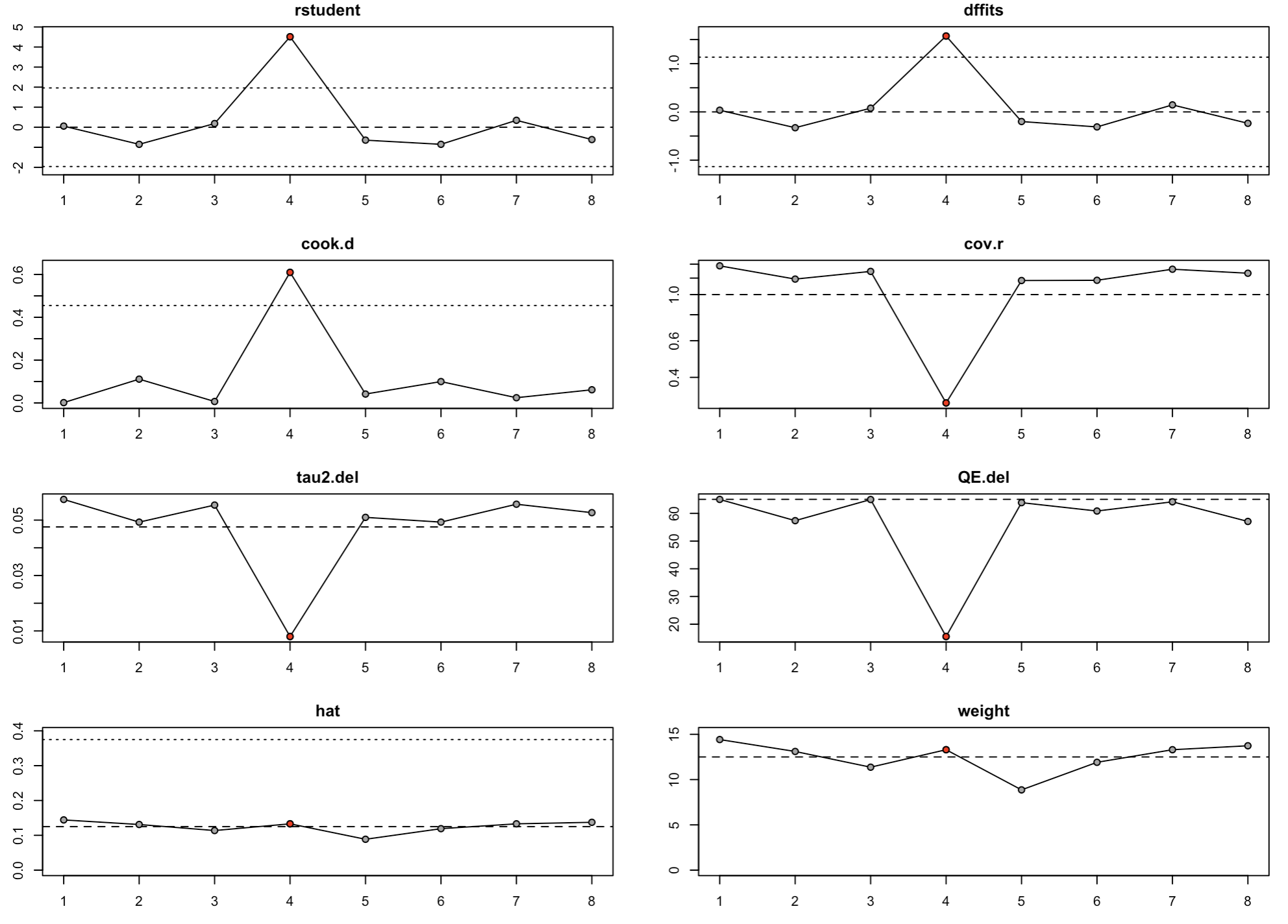


- - 1. **Achievement emotions and L2 learning**

**Figure S2.**

Diagnostic graphs of the influence of studies on anxiety and L2 learning.

**
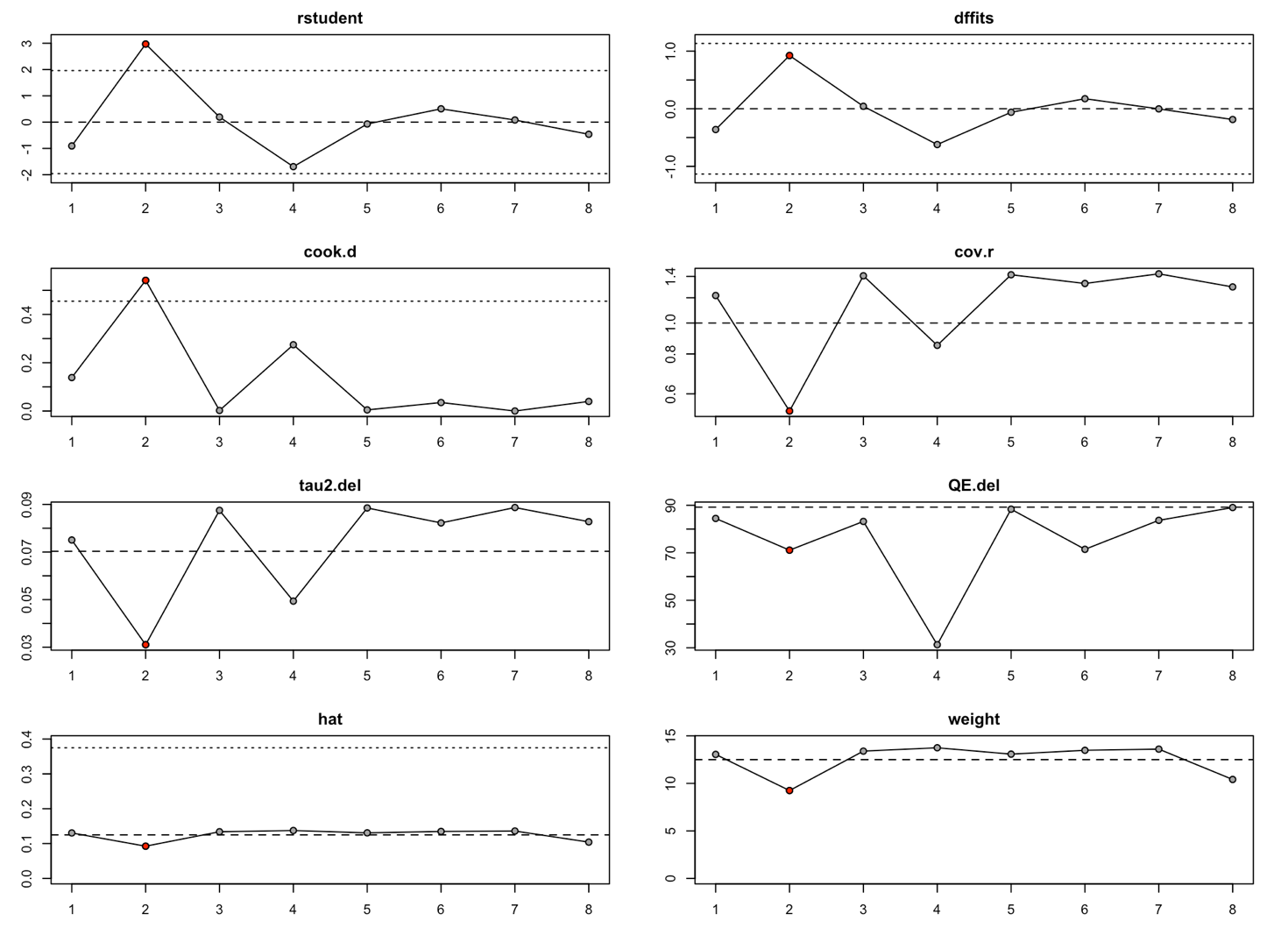
**
