# Supplementary material for: A systematic review and meta-analysis on achievement emotions, working memory and student-teacher relationship during second language learning in primary school
Source: PLoS One. 2026 May 26;21(5):e0350119. doi: 10.1371/journal.pone.0350119 (PMC13210231; doi:10.1371/journal.pone.0350119)
Supplement: Attachment — Submitted filename: rebuttal letter.docx [file pone.0350119.s004.docx]

PONE-D-25-58700

A systematic review and metanalysis on achievement emotions, working memory and student-teacher relationship during second language learning in primary school.

PLOS ONE

Dear Dr. Trotta,

Thank you for submitting your manuscript for review by PLOS ONE. After careful consideration, we have decided that your manuscript does not meet our standards for systematic reviews. We are therefore rejecting your manuscript.

PLOS ONE considers systematic reviews and meta-analyses for publication; however, these submissions must address a clear and defined research question; conduct a systematic and comprehensive literature review; and use clearly reported, reproducible, and systematic methods to identify, select, and critically appraise relevant research. Our author guidelines for systematic reviews are at https://journals.plos.org/plosone/s/submission-guidelines#loc-systematic-reviews-and-meta-analyses.

In the case of your submission, we note that language restrictions are imposed on the literature searches without adequate justification. There is increasing recognition that exclusion of studies published in other languages can increase the risk of bias in systematic reviews. We feel that any restrictions should be justified based on scientific reasons rather than resource constraints. As a result, we have concerns about the comprehensiveness of the review and the validity of the conclusions.

As a result of these concerns, we cannot consider the manuscript for publication at PLOS ONE at this time. However, if you are able to address the concerns discussed above, we may be able to consider a revised version of the manuscript. This would however require a substantial amount of work and thus, would be considered as a new submission. If you are able to revise the manuscript to respond to the concerns noted above, please refer to the original submission in the covering letter and accompany the submission by a rebuttal letter that responds to the comments made on the original manuscript.

I am sorry that we cannot be more positive on this occasion, but I hope you understand the reasons for this decision.

Kind regards,

Asmita Karmakar, PhD

Editorial Office

PLOS ONE

Dear Dr. Karmakar and the Editorial Team,

We would like to thank you for your consideration of our manuscript. We carefully reviewed your comments and fully acknowledge your concern regarding the language restrictions applied during the literature search.

The decision to include only articles written in English and Italian was based on the linguistic competencies of the research team conducting the systematic review and meta-analysis. However, we agree that excluding studies published in other languages can increase the risk of bias and potentially limit the comprehensiveness of the review. We also recognize that current technological tools can significantly reduce language barriers.

As illustrated in the PRISMA flowchart of the literature search and screening process (Figure 1 of our manuscript), our search identified three scientific articles written in languages other than English or Italian. To address your concern, we employed artificial intelligence–based translation tools to obtain literal translations of these non-English/Italian studies.

After reviewing the translated versions, we found several reasons that justified their exclusion, irrespective of the language of publication. Specifically, one of the three articles was not accessible (we contacted the authors but did not receive a response), and the remaining two did not address any of the variables of interest in our study (working memory, achievement emotions, or student-teacher relationship).

Consequently, we added the following clarification to line 271 of the revised manuscript:

“Regarding studies written in languages other than those included among the inclusion criteria, it is known that meta-analyses with language restrictions tend to overestimate treatment effects compared to those including studies in all available languages, although they yield more precise results (Moher et al., 1999; Morrison et al., 2021; Dobrescu et al., 2021). As illustrated in the PRISMA flowchart of the literature search and screening process (Figure 1), our search identified three scientific articles written in languages other than English and Italian, one of which was unavailable (requested from the authors but not received). To eliminate any potential bias, we used artificial intelligence to obtain literal translations of the remaining two articles. Regardless of language restrictions, neither of these two studies addressed any of our variables of interest (WM, AEs, STR). Therefore, their exclusion from the data analysis was confirmed.”

We hope that this clarification and the following revisions may address the concerns raised and demonstrate our commitment to methodological rigor and transparency.

We thank you again for your valuable feedback and for giving us the opportunity to improve our manuscript.

Kind regards,

Eugenio Trotta, PhD

on behalf of all co-authors
