# Supplementary material for: A systematic review and meta-analysis on achievement emotions, working memory and student-teacher relationship during second language learning in primary school
Source: PLoS One. 2026 May 26;21(5):e0350119. doi: 10.1371/journal.pone.0350119 (PMC13210231; doi:10.1371/journal.pone.0350119)
Supplement: Attachment — Submitted filename: PONE-D-25-59942-Response letter.docx [file pone.0350119.s005.docx]

**Point-by-point response letter** **PONE-D-25-59942: “A systematic review and metanalysis on achievement emotions, working memory and student-teacher relationship during second language learning in primary school”.**

We thank the Academic Editor and the Reviewers for the time dedicated to our manuscript and for the constructive comments provided. Below, we present the Journal Requirements and the Reviewers’ comments to the Authors in black. The Authors’ responses are in blue. All changes in the revised manuscript are tracked with the “track changes” option.

**Journal Requirements.**

When submitting your revision, we need you to address these additional requirements.

1. Please ensure that your manuscript meets PLOS ONE’s style requirements, including those for file naming. The PLOS ONE style templates can be found at <https://journals.plos.org/plosone/s/file?id=wjVg/PLOSOne_formatting_sample_main_body.pdf> and <https://journals.plos.org/plosone/s/file?id=ba62/PLOSOne_formatting_sample_title_authors_affiliations.pdf>

We confirm that our manuscript meets PLOS ONE’s style requirements. However, we cited Figures as “Figure” (e.g., Figure 1; Figure 2; etc.) instead of “Fig” as suggested by the manuscript body formatting guidelines. We modified it.

2. We note that the grant information you provided in the ‘Funding Information’ and ‘Financial Disclosure’ sections do not match. When you resubmit, please ensure that you provide the correct grant numbers for the awards you received for your study in the ‘Funding Information’ section.

We apologize for the inconvenience. It was unintentional. As request, we are going to report as follow, when we resubmit:

*This work was supported by the “Learning English as a second language in primary school: an investigation of metacognitive and socio-emotional factors on children’s academic success and wellbeing - SELF_ENG”, Research Project of Relevant National Interest 2022 (PRIN), part of the National Recovery and Resilience Plan (PNRR) also known as “Italia Domani”, and component of the European Union’s Next Generation EU recovery program (Protocol n. P2022TT8LH).*

3. Please note that funding information should not appear in any section or other areas of your manuscript. We will only publish funding information present in the Funding Statement section of the online submission form. Please remove any funding-related text from the manuscript.

As request, we removed any funding-related text from the manuscript. Specifically, we removed the reference for the awards we received for our study (page 1, line 21) and the “Funding sources” section (page 23, line 599).

4. If the reviewer comments include a recommendation to cite specific previously published works, please review and evaluate these publications to determine whether they are relevant and should be cited. There is no requirement to cite these works unless the editor has indicated otherwise.

No reviewer comments included a recommendation to cite specific previously published works.

5. Please review your reference list to ensure that it is complete and correct. If you have cited papers that have been retracted, please include the rationale for doing so in the manuscript text, or remove these references and replace them with relevant current references. Any changes to the reference list should be mentioned in the rebuttal letter that accompanies your revised manuscript. If you need to cite a retracted article, indicate the article’s retracted status in the References list and also include a citation and full reference for the retraction notice.

We confirm that our reference list is complete and correct. We did not identify cited papers that have been retracted.

**Comments to the Authors from Reviewer 1.**

Overall, the manuscript is structured with a clear and easy-to-follow flow, from the background, objectives, PRISMA methods, bias assessment, narrative synthesis, and meta-analysis, to the discussion and limitations. The English is generally up to academic standards but still needs refinement to be more consistent and unambiguous, as PLOS ONE does not perform post-acceptance language editing.

We thank Reviewer 1 for appreciating our work. As suggested, we refined the English of our manuscript to be more consistent and unambiguous with academic standards.

Improvement Notes:

- Unify terminology, particularly using a single, consistent form for “meta-analysis” and avoiding variations such as “metanalysis.”

We thank Reviewer 1 for the suggestion. We have consolidated the terminology with the term “meta-analysis.”

- Correct several grammatical errors and unnatural phrasing and break up long sentences for better readability.

Thank you for the suggestion. We revised the manuscript to improve grammatical accuracy, reduce long sentences, and enhance readability.

- Ensure abbreviations (WM, AEs, STR, L1, L2) are defined once at the outset and used consistently.

We ensure that all abbreviations are defined upon first use and used consistently.

- Clean up the formatting of lists and tables to avoid skipped numbering or layout artifacts.

Thank you for the suggestion. We revised the manuscript to ensure consistent layout, correct numbering, and the removal of any spacing or formatting artifacts.

- Exercise caution in claims, especially for STR, as quantitative evidence is limited and relies heavily on qualitative synthesis.

We thank Reviewer 1 for the observation. Indeed, the evidence regarding STR is limited to qualitative data. We revised the manuscript, adopting more cautious language in interpreting these results.

- Furthermore, the authors mention the use of AI to translate non-English or non-Italian articles. Translation procedures and verification should be explained concisely for transparency.

We thank Reviewer 1 for the comment. As suggested, we clarified in the manuscript the translation and review procedures for articles not available in English or Italian. Briefly, translations were obtained using two independent tools, and the resulting texts were subsequently checked by the research team for consistency with the original structure and key methodological information.

- Additionally, explain more explicitly how to detect and handle potential sample duplication in follow-up studies. Authors should also ensure a complete statement of data availability and supporting materials, including the search strategy and dataset extraction.

As suggested, we detailed how we handled papers with the same sample in "Description of the studies included" section. All data analysis and supporting materials, including the search strategy and dataset extraction, were previously shared within the Supporting Information. We forgot to mention it in the main manuscript. We thank the Reviewer 1 for the suggestion, giving us the opportunity to report it (see "Literature Search and search strategy" section).

In conclusion, the manuscript is clear enough, but requires light to moderate editing for terminology consistency, minor grammar corrections, and formatting refinement before publication.

We thank the Reviewer 1 for the time dedicated to our manuscript and for the constructive comments provided.

**Comments to the Authors from Reviewer 2.**

This systematic review and meta-analysis addresses a timely and important topic in educational psychology—the interplay between cognitive (working memory), emotional (achievement emotions), and socio-affective factors (student-teacher relationship) in second language learning during primary school. The manuscript makes a valuable contribution to the field by synthesizing evidence across 19 primary studies with over 5,300 participants.

Strengths:

- The research addresses a significant gap by examining how multiple factors interact in L2 learning, moving beyond isolated variable approaches
- Methodological rigor is evident in the systematic review process and meta-analytic techniques
- Findings confirm expected associations (working memory positively correlates with L2 outcomes) while also revealing nuanced patterns (differential effects of anxiety versus enjoyment)
- The qualitative synthesis of student-teacher relationship research provides valuable insights about the protective function of emotional closeness, particularly for anxious learners
- The authors appropriately acknowledge limitations and suggest directions for future research

We thank Reviewer 2 for appreciating our work.

Minor Revisions Suggested:

- Consider adding a brief discussion of implications for educational practice, particularly how teachers might leverage positive relationships to mitigate anxiety

We thank Reviewer 2 for his suggestion. We expanded the Discussion section by adding a practical example of how teachers can help reduce anxiety and support their students’ cognitive competences by promoting an emotionally safe and engaging environment.

- A PRISMA flowchart would enhance transparency of the study selection process
  We agree with Reviewer 2. We previously reported our PRISMA flowchart of the literature search and screening process as Fig 1. Since the manuscript body formatting guidelines state “figures should be uploaded separately as individual files”, you can consult our PRISMA flowchart inside the file named “Figures”.
- The conclusion could be strengthened by explicitly connecting findings to the broader context of multilingual education policies.

We thank Reviewer 2 for the suggestion. As suggested, we integrated our findings into multilingual education policies.

Overall, this well-conducted review advances our understanding of the multidimensional nature of L2 learning in primary school settings and deserves publication.

We thank the Reviewer 2 for the time dedicated to our manuscript and for the constructive comments provided.
