# Supplementary material for: A systematic review and meta-analysis on achievement emotions, working memory and student-teacher relationship during second language learning in primary school
Source: PLoS One. 2026 May 26;21(5):e0350119. doi: 10.1371/journal.pone.0350119 (PMC13210231; doi:10.1371/journal.pone.0350119)
Supplement: Attachment — Submitted filename: PONE-D-25-59942R1- 2ndResponse letter.docx [file pone.0350119.s006.docx]

**Point-by-point response letter** **PONE-D-25-59942R1: “A systematic review and meta-analysis on achievement emotions, working memory and student-teacher relationship during second language learning in primary school”.**

We thank the Academic Editor and the Reviewers for the time dedicated to our manuscript and for the constructive comments provided. Below, we present the Reviewers’ comments to the Authors in black. The Authors’ responses are in blue. All changes in the revised manuscript are tracked with the “track changes” option.

**Comments to the Authors from Reviewer 1.**

This manuscript presents a systematic review and meta-analysis examining the relationship between working memory, achievement emotions, and student–teacher relationships in second language (L2) learning among primary school students. The topic is relevant and timely, as understanding the interaction between cognitive, emotional, and contextual factors is important for improving language learning outcomes. Overall, the manuscript demonstrates potential to contribute to the literature in educational psychology and second language acquisition.

The study applies an appropriate systematic review methodology and follows PRISMA guidelines for study selection. The use of meta-analytic techniques, including random-effects models, heterogeneity analysis, and publication bias assessment, indicates an effort to ensure methodological rigor. The integration of three important constructs (working memory, achievement emotions, and student–teacher relationships) provides a multidimensional perspective that is valuable for both researchers and practitioners.

However, several issues should be addressed to improve the scientific quality and clarity of the manuscript.

We thank Reviewer 1 for the time dedicated to our manuscript and for appreciating our work.

First, although the statistical analyses are generally appropriate, the number of studies examining student–teacher relationships appears limited, which may affect the strength and generalizability of the conclusions related to this variable. The authors are encouraged to discuss this limitation more explicitly and clarify how it may influence interpretation of the results.

We thank Reviewer 1 for the comment. We agree that the limited number of studies investigating the student–teacher relationship (STR) represents a constraint for the strength and generalizability of the conclusions. As reported in the first limit (page 29, row 548), only four studies addressed STR, and only two provided quantitative data suitable for meta-analysis. For this reason, we treated the evidence on STR exclusively at a qualitative level and explicitly framed these findings as exploratory. To address this concern, we strengthened the discussion of this limitation in the manuscript by clarifying that:

- the small number of studies prevents robust generalization of the STR findings;
- conclusions regarding STR should be interpreted with caution;
- further research is needed to provide more solid and generalizable evidence on this variable.

Second, the manuscript would benefit from clearer reporting regarding data availability. According to PLOS data policy, all data underlying the findings should be fully accessible either within the manuscript, supporting information files, or a public repository. The authors should provide explicit information about where the extracted dataset, effect size calculations, and analysis files can be accessed.

We totally agree with Reviewer 1 on the importance of transparency and data availability. As already indicated in the manuscript (see “Literature search and search strategy”, line 195), all data underlying the findings are fully accessible in the Supporting Information files. Specifically:

- The complete search strategy (including research question development, use of synonyms, and Boolean operators across all databases) is provided in S1 File “Additional information for Method section”;
- All effect size calculations, as well as sensitivity analyses, are reported in S2 File “Additional information on quantitative analyses”.

Furthermore, in line with PLOS data-sharing policies, the study was preregistered on a public repository (Open Science Framework). For the purposes of double-blind peer review, an anonymous access link is currently provided in the manuscript; this will be replaced with a fully accessible public link upon publication.

Given the extent and technical detail of the search strategy and quantitative analyses, these materials are not included in full within the main manuscript but are entirely available in the supplementary files to ensure transparency and reproducibility. To further improve clarity, we strengthened the description of data availability in the manuscript.

Third, the manuscript requires careful language editing to improve clarity and readability. Several grammatical issues, repeated phrases, and awkward sentence structures are present throughout the text. Professional proofreading is recommended to ensure the manuscript meets the standard of academic English expected for publication.

We thank Reviewer 1 for the recommendation. We declare that we used ChatGPT (OpenAI, San Francisco, CA, USA) to improve the English language and the writing quality. After using it, we reviewed and edited the content as needed and took full responsibility for the content of the publication.

Additionally, the authors may consider improving the consistency of terminology and providing clearer explanations of the theoretical framework linking cognitive and emotional factors in L2 learning. Clarifying the rationale for inclusion criteria and providing more detailed justification for methodological choices would further strengthen the manuscript.

We improved clarity, refined sentence structure, ensured terminological consistency, and strengthened the theoretical framework by explicitly highlighting the interaction between cognitive, emotional, and relational factors throughout the Introduction.

Despite these concerns, the study addresses an important research topic and applies appropriate methodological procedures. With revisions focusing on language clarity, transparency of data availability, and clearer discussion of limitations, the manuscript has the potential to make a meaningful contribution to the field.

No concerns related to research ethics, plagiarism, or duplicate publication were identified.

Overall recommendation: minor revision.

We thank Reviewer 1 for the time dedicated to our manuscript and for appreciating our work.

**Comments to the Authors from Reviewer 2.**

This is an excellent study. The methods are rigorous, the analyses are appropriate, and the conclusions are well supported. I have no further suggestions—congratulations on this valuable contribution.

We thank Reviewer 2 for the time dedicated to our manuscript and for appreciating our work.
